# Temporally divergent regulatory mechanisms govern neuronal diversification and maturation in the mouse and marmoset neocortex

**DOI:** 10.1038/s41593-022-01123-4

**Published:** 2022-08-01

**Authors:** Wen Yuan, Sai Ma, Juliana R. Brown, Kwanho Kim, Vanessa Murek, Lucia Trastulla, Alexander Meissner, Simona Lodato, Ashwin S. Shetty, Joshua Z. Levin, Jason D. Buenrostro, Michael J. Ziller, Paola Arlotta

**Affiliations:** 1 Department of Stem Cell and Regenerative Biology, Harvard University, Cambridge, MA USA; 2 Klarman Cell Observatory, Broad Institute of MIT and Harvard, Cambridge, MA USA; 3 Department of Biology and Koch Institute, Massachusetts Institute of Technology, Cambridge, MA USA; 4 Stanley Center for Psychiatric Research, Broad Institute of Harvard and MIT, Cambridge, MA USA; 5 Max Planck Institute of Psychiatry, Munich, Germany; 6 Department of Genome Regulation, Max Planck Institute for Molecular Genetics, Berlin, Germany; 7 Broad Institute of MIT and Harvard, Cambridge, MA USA; 8 Department of Biomedical Sciences, Humanitas University, Pieve Emanuele, Milan, Italy; 9 IRCCS Humanitas Research Hospital, Rozzano, Milan, Italy; 10 Department of Psychiatry, University of Münster, Münster, Germany

**Keywords:** Cell type diversity, Epigenetics in the nervous system, Transcriptomics

## Abstract

Mammalian neocortical neurons span one of the most diverse cell type spectra of any tissue. Cortical neurons are born during embryonic development, and their maturation extends into postnatal life. The regulatory strategies underlying progressive neuronal development and maturation remain unclear. Here we present an integrated single-cell epigenomic and transcriptional analysis of individual mouse and marmoset cortical neuron classes, spanning both early postmitotic stages of identity acquisition and later stages of neuronal plasticity and circuit integration. We found that, in both species, the regulatory strategies controlling early and late stages of pan-neuronal development diverge. Early postmitotic neurons use more widely shared and evolutionarily conserved molecular regulatory programs. In contrast, programs active during later neuronal maturation are more brain- and neuron-specific and more evolutionarily divergent. Our work uncovers a temporal shift in regulatory choices during neuronal diversification and maturation in both mice and marmosets, which likely reflects unique evolutionary constraints on distinct events of neuronal development in the neocortex.

## Abstract (web summary)

The mechanisms underlying neuron specification and maturation are unclear. Here the authors provide an integrated epigenomic and transcriptomic analysis of mouse and marmoset neocortical neuronal classes. Pan-neuronal programs active during early development are more evolutionary conserved but not neuron-specific, whereas pan-neuronal programs active during later stages of maturation are more neuron- and species-specific.

## Main

The mammalian cerebral cortex contains a great diversity of neurons that differ in their connectivity, neurotransmitter usage, morphology, gene expression and electrophysiological properties^1^. Although recent work has uncovered the molecular states that define different classes of terminally differentiated neurons in the adult brain^2–11^, profiling of cortical neurons over developmental time courses has been limited and largely confined to transcriptional profiling of a few embryonic and neonatal stages^12–15^ or of adult timepoints^10,13,16–24^. There is not yet a holistic picture of the molecular dynamics and regulatory landscapes of individual cortical neuron classes, or any other class of mammalian central neurons, over extended trajectories of cell identity acquisition and maturation during postnatal life. This has precluded in-depth understanding of the molecular strategies used by neurons to acquire their identity, to mature and to wire. Similarly, how such molecular strategies might vary across species has not been addressed.

Genomic approaches have been instrumental to studying the molecular logic of cellular differentiation and cell type identity acquisition in the brain^10,25–29^ and other tissues^30,31^. Assay for transposase-accessible chromatin using sequencing (ATAC-seq) methods have shown that the number of active enhancer regions varies across development in different cell types; for example, during differentiation of helper T cells, enhancer usage decreases during maturation^32^, whereas cardiomyocytes employ a constant number of enhancers across differentiation^33^. During B cell maturation, the number of active enhancers decreases as they mature from hematopoietic stem cells to terminal cell types^34^. Despite progress, the regulatory logic that accompanies cell-type-specific differentiation across tissues remains understudied. This is particularly true for the central nervous system, where it is unclear whether common regulatory strategies controlling the development of the great diversity of neural cell types exist.

Here we provide a comprehensive single-cell dataset of defined transcriptional and epigenomic changes in different classes of postmitotic cortical neurons over a time course spanning perinatal ages to adulthood, in both mouse and marmoset. We uncover previously unappreciated divergence in pan-neuronal regulatory mechanisms governing early stages of neuronal development versus later stages of neuronal circuit formation. These distinct regulatory modes represent a common strategy across all cortical neuron types and are conserved between mice and non-human primates.

## Results

### Early and late stages of cortical pyramidal neuron postmitotic development use divergent regulatory programs

Even after becoming postmitotic, cortical neurons undergo extensive development, from establishment of subtype identity to postnatal refinement of terminally differentiated features. It is largely not understood whether regulatory strategies at play during postmitotic development remain constant over postnatal life.

**Fig. 1:**
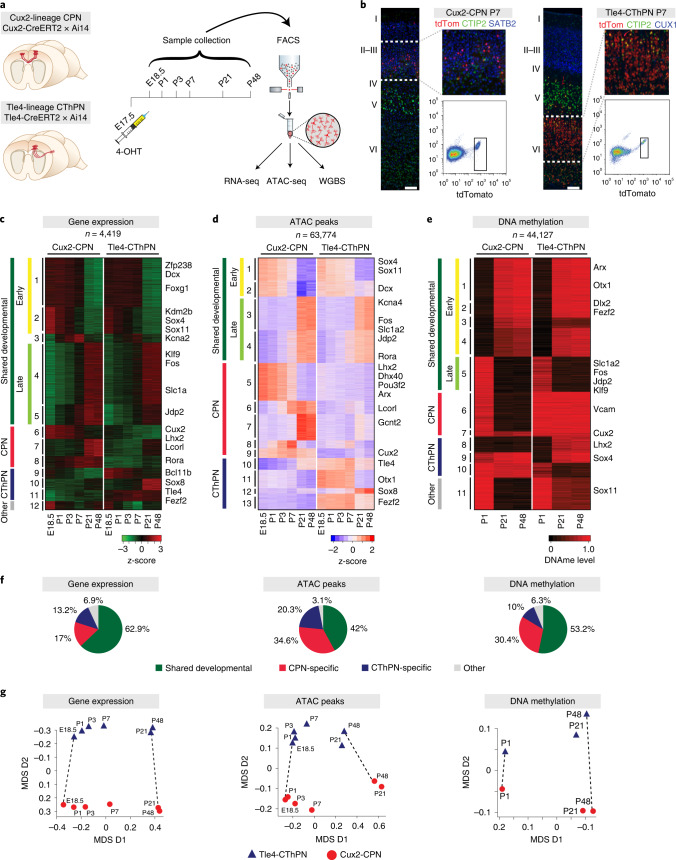
Profiling of genetically defined cortical pyramidal neuron classes. **a**, Schematic of experimental design. **b**, Representative coronal sections (*n* ≥ 3 biological replicates) showing correct laminar location of tdTomato^+^ cells in the somatosensory cortex and FACS plots of Tle4-CThPNs and Cux2-CPNs at P7. Scale bar, 100 μm. Also see Extended Data Fig. 1. **c**–**e**, Developmental dynamics of differentially expressed genes (**c**), differentially enriched ATAC-seq peaks (**d**) and DMRs (**e**). **f**, Fraction of dynamic features classified into each overall category. **g**, 2D MDS plots for each modality.

**Extended Data Fig. 1:**
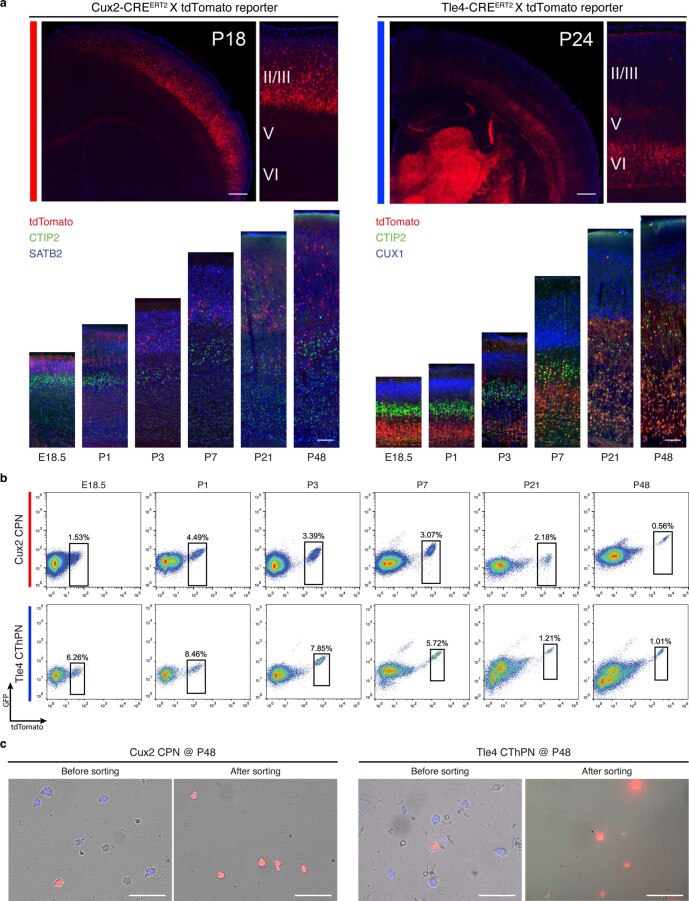
Isolation of genetically-labelled CPNs and CThPNs. **a**, Representative coronal sections (*n* ≥ 3 biological replicates) showing correct laminar location of tdTomato^+^ cells in the somatosensory cortex at the different developmental stages. Upper panels, scale bar 500 μm. Lower panels, scale bar 100 μm. **b**, Representative FACS results for purification of Cux2 CPN and Tle4 CThPN populations at the different developmental stages. Boxed areas indicate tdTomato^+^ cells collected for profiling. The GFP channel (empty) is used for evaluation of background signal. **c**, Representative images of pre-and post-sorting Tle4 and Cux2 neuronal populations at P48, showing good purity and viability even at this older age (*n* ≥ 3 biological replicates). Scale bar, 50 μm.

To understand these regulatory strategies, we first applied inducible Cre mouse lines to examine two major classes of neocortical pyramidal neurons: *Cux2*-lineage^35^ layer 2/3 (L2/3) callosal projection neurons (CPNs)^1^, which are involved in associative functions and are the most recently evolved population of cortical neurons (henceforth, Cux2 CPNs), and *Tle4*-lineage^36^ layer 6 (L6) corticothalamic projection neurons (CThPNs)^1^, which are responsible for integration of sensory and motor information (henceforth, Tle4 CThPNs). We isolated these neuronal subtypes across a time course spanning the acquisition of class-specific neuronal identity and early neuronal development (embryonic day (E) 18.5 and postnatal day (P) 1, P3 and P7), through periods of cortical plasticity, neuronal maturation and integration into cortical circuits (P21 and P48) (Fig. 1a, Extended Data Fig. 1 and Supplementary Fig. 1). Labeled neurons from dissected somatosensory and motor cortex (ten animals per library, five male and five female, from two litters) were isolated by fluorescence-activated cell sorting (FACS) and profiled in bulk for gene expression by RNA sequencing (RNA-seq), for DNA methylation (DNAme) by whole-genome bisulfite sequencing (WGBS) and for open chromatin by ATAC-seq. Two biological replicates were performed for each age and neuron type for each assay.

We identified features (transcribed genes, differentially accessible chromatin peaks and differentially methylated regions (DMRs)) that were dynamic over age or between cell types (examples in Supplementary Fig. 2) and applied k-means clustering to group features with similar patterns (Fig. 1c–e, Supplementary Fig. 3 and Supplementary Tables 1–3). For all datasets, 40–60% of dynamic features were assigned to clusters that were associated with developmental stage and independent of neuronal subtype (Fig. 1c–f). These shared, developmentally regulated clusters fell into two major categories: those predominantly active (transcriptionally upregulated, accessible or hypomethylated) at embryonic and/or early postnatal ages (E18.5 to P7; early developmental, yellow bars in Fig. 1c–e) and those predominantly active at weaning and older ages (P21 to P48; late developmental, light green bars in Fig. 1c–e).

A smaller proportion of dynamic features showed neuron class-specific patterns. As expected, these clusters included known molecular markers of CPNs and CThPNs^37,38^ (Fig. 1c–e). Notably, although class-specific clusters accounted for only 23% of dynamic transcriptional features, they comprised 34% (DNAme) to 45% (ATAC) of dynamic epigenetic features (Fig. 1f), indicating higher neuronal subtype specificity of epigenetic changes, in agreement with findings that epigenetic signatures may be particularly powerful in discriminating neuronal subclasses^16,39^.

**Extended Data Fig. 2:**
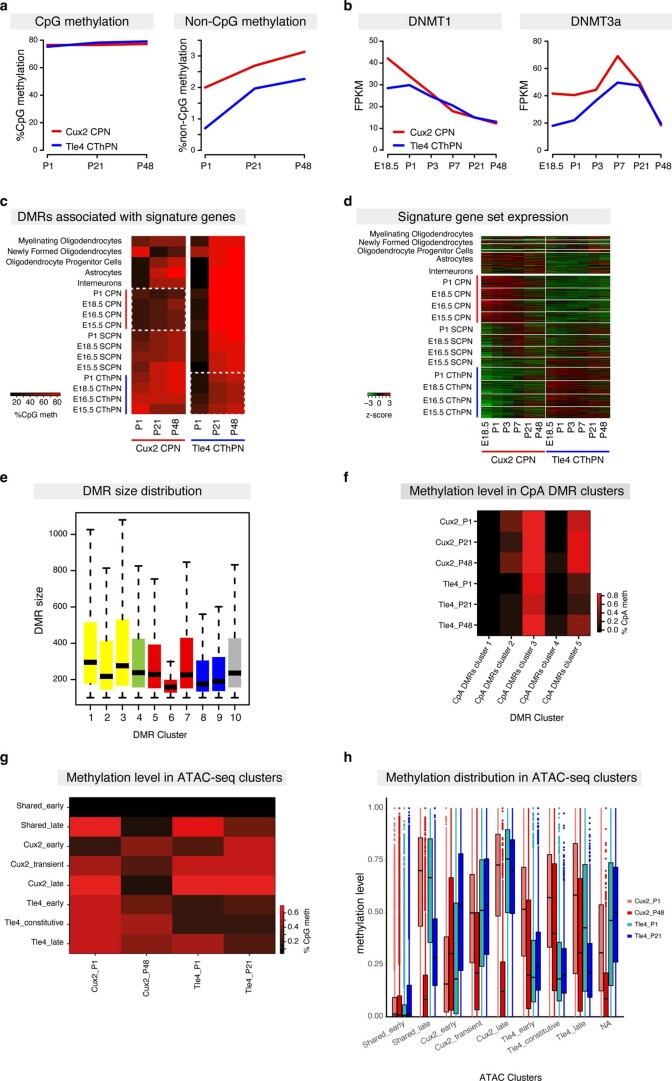
DNA methylation at dynamic loci. **a**, Global DNA methylation levels at CpG (left) and non-CpG sites (right) across ages. **b**, Average expression of differentially expressed DNA methyltransferases across ages. **c**, Median methylation levels of all DMRs within 100 kb of signature genes for the specified cell types at each age. Boxes indicate gene sets corresponding to earlier ages of the same cell fate. **d**, Normalized gene expression patterns, shown as Z-scores, for the sets of signature genes used in panel **c**. **e**, Size distribution of DMRs within each cluster. Bar: median; box: 25–75th quantile; whisker: 1.5 x inter-quartile range. For size of clusters, see Supplementary Fig 3c. **f**, Average CpA methylation levels across 11,150 CpA DMRs clustered into 5 distinct clusters across developmental time and PN subtypes. **g**, Average methylation levels for ATAC-seq regions with overlapping DNAme data for each ATAC-seq cluster (*y* axis) across developmental time and neuronal subtype (x-axis) based on bulk ATAC-seq data. **h**, Methylation level distribution (*y* axis) for ATAC-seq regions with overlapping DNAme data for each ATAC-seq cluster (*x* axis) across developmental time and neuronal subtype (colored boxplots) based on bulk ATAC-seq data. Box and midline: 25th, 50th and 75th percentiles; whiskers: 1.5 interquartile range from box. Dots indicate data points outside this range.

We applied multi-dimensional scaling (MDS) to visualize the relative distance between the high-dimensional transcriptional and epigenetic landscapes over time. Despite widespread changes over development, these two classes of neurons showed only limited changes in overall similarity with time in either gene expression or open chromatin profiles (Fig. 1g and Supplementary Fig. 3b). In contrast, the DNA methylation landscape became more divergent between neuronal subtypes over postnatal life (*P* < 0.03329, one-sided *t*-test; Fig. 1g and Supplementary Fig. 3b). This increase was not associated with global changes in methylation or expression of DNA methylases (Extended Data Fig. 2a,b) but, rather, with changes in distribution patterns across the genome. DNA methylation increased over time at genes and gene regulatory elements (GREs, inferred from open chromatin sites) characteristic of other cell types and earlier developmental stages (Extended Data Fig. 2c,d), consistent with its known role in stabilizing silencing of inappropriate transcriptional programs^40^. Similarly, analysis of CpA methylation (the dominant form of non-CpG methylation in mammals) identified 11,150 DMRs across cell types and developmental time, although with small effect sizes (Extended Data Fig. 2f). Notably, CpA methylation exhibited dynamic patterns that were similar to those found for CpG methylation but showed only minimal overlap with CpG DMRs (<10%).

Next, to identify strategies for genome regulation that are common to pyramidal neuron classes across developmental time, we examined clusters whose temporal dynamics were shared between both cell types (shared-early and shared-late clusters; Fig. 1c–e). This uncovered a pronounced temporal transition in regulatory dynamics, across multiple modalities, between shared programs of early (E18.5 to P7) versus late (P21 to P48) postmitotic neuronal development.

**Extended Data Fig. 3:**
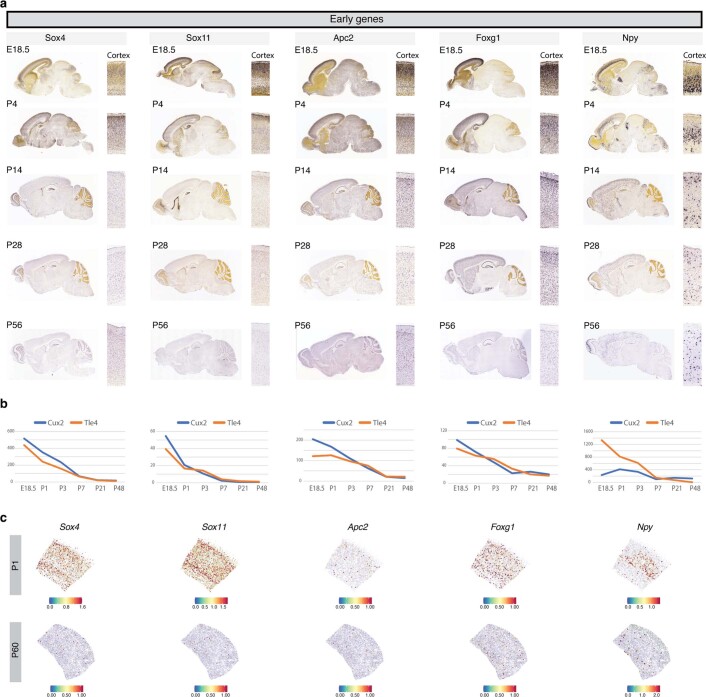
Validation of temporal patterns of early gene expression using external datasets. **a**, Expression of selected genes in the shared-early cluster at (from top to bottom) E18.5, P4, P14, P28 and P56, in the mouse brain in situ hybridization (ISH) database from the Allen Institute, showing the whole brain and an enlargement of the cortex. **b**, Gene expression values for the corresponding genes in the Cux2-CPN (red) and Tle4-CThPN (blue) RNA-seq data. Units: fragments per kilobase of exon per million reads mapped (FPKM). **c**, Gene expression level in P1 and P60 mouse cortex by Slide-seq^42^.

**Extended Data Fig. 4:**
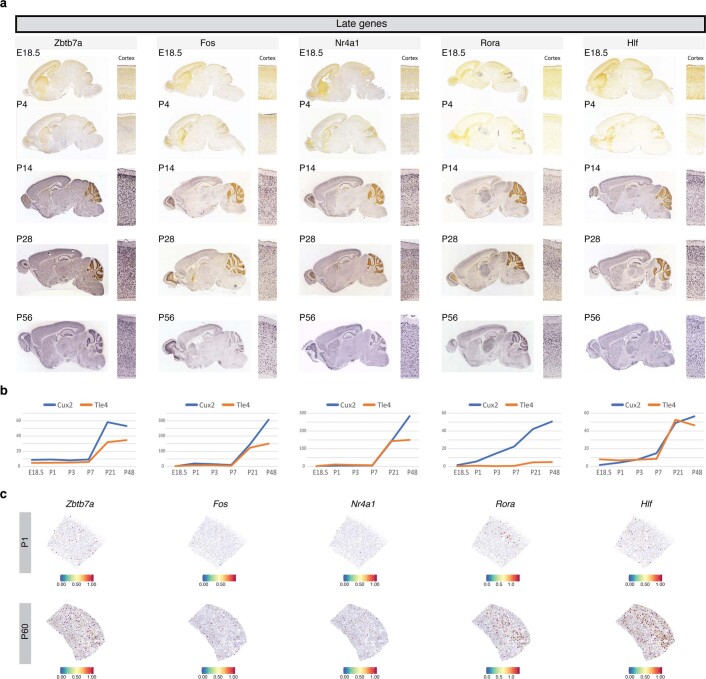
Validation of temporal patterns of late gene expression using external datasets. **a**, Expression of selected genes in the shared-late cluster at (from top to bottom) E18.5, P4, P14, P28 and P56, in the mouse brain in situ hybridization (ISH) database from the Allen Institute, showing the whole brain and an enlargement of the cortex. **b**, Gene expression values for the corresponding genes in the Cux2-CPN (red) and Tle4-CThPN (blue) RNA-seq data. Units: fragments per kilobase of exon per million reads mapped (FPKM). **c**, Gene expression level in P1 and P60 mouse cortex by Slide-seq^42^.

The shared-early clusters contained genes typical of early differentiation events, such as *Sox4*, *Sox11* and *Apc2*, whereas the shared-late clusters contained genes controlling later processes such as synaptic function, including *Egr3*, *Syp* and *Nefm*. We validated temporal expression patterns in the cortex for selected example genes using the Allen Institute mouse brain in situ hybridization (ISH) database^41^ (Extended Data Figs. 3a,b and 4a,b) and a Slide-seq spatial transcriptomics dataset of young and adult mouse cortex^42^ (Extended Data Figs. 3c and 4c).

To examine whether these temporal patterns were conserved in humans, we leveraged human developing brain transcriptomic data from BrainSpan^43^. About 85% of the genes in both the shared-early (1,129/1,334) and shared-late (1,234/1,446) gene clusters had orthologs that were expressed in the human data, and most of these showed similar temporal dynamics as in mouse (Supplementary Fig. 4).

**Fig. 2:**
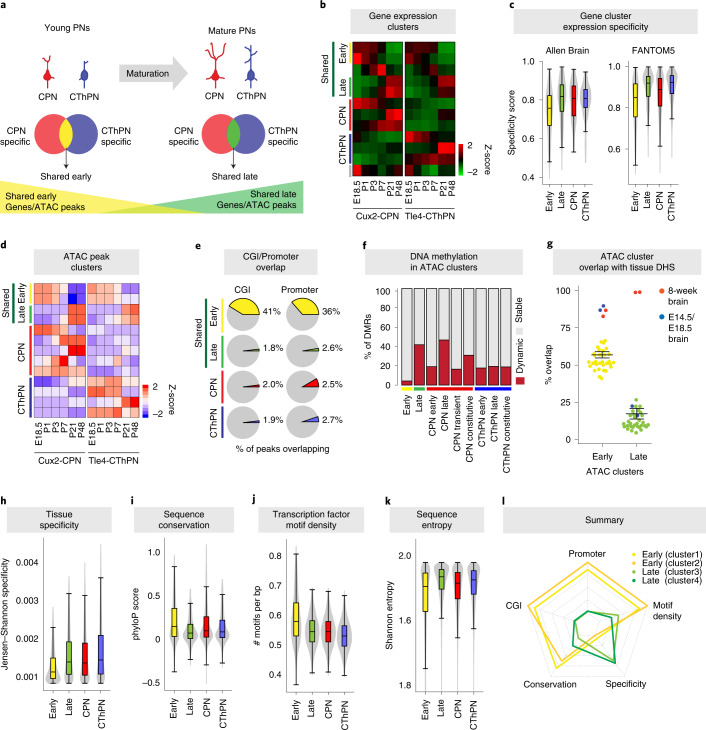
Divergent regulatory principles of early and late stages of neuronal development. **a**, Schematic of analyses. **b**, Summary of gene expression clusters from Fig. 1c. **c**, TF expression specificity within the brain for TFs in different categories of cluster, from expression data from the Allen Brain Atlas (left). Higher values indicate more specific expression. TF expression specificity across 397 mouse cell types from the FANTOM5 project (right). Box and midline: 25th, 50th and 75th percentiles; whiskers: 1.5× interquartile range from box. **d**, Summary of ATAC peak clusters from Fig. 1d. **e**, Fraction of open chromatin regions (ATAC-seq peaks) in different categories of clusters that overlap CGIs or annotated promoter regions. **f**, Fraction of DMRs in ATAC clusters of different categories that are static or dynamic over the time course. **g**, Fraction of ATAC-seq peaks from different classes of ATAC clusters that overlap DNAse hypersensitivity sites (DHSs) across 35 cell types from the mouse ENCODE project. Adult (8-week) whole-brain and telencephalon samples highlighted in red; embryonic (E14.5 and E18.5) brain samples are highlighted in blue. Also see Extended Data Fig. 5f. Error bars: mean ± s.e.m. **h**, Open chromatin specificity across ATAC peak clusters (*n*_Early_ = 10,488, *n*_Late_ = 17,570), as the concordance of ATAC peaks with an scATAC-seq panel of 85 mouse primary tissues and cell types^17^. Box plot as in **c**. **i**, Distribution of phyloP sequence conservation across all placental mammals for early developmental and late developmental ATAC clusters. Box plot as in **c**. **j**, Density of known TF binding motifs within ATAC clusters of different types. Box plot as in **c**. **k**, Average sequence entropy within ATAC clusters of different types. Box plot as in **c**. **l**, Summary of characteristics from **c** and **g**–**j** for each of the shared developmental ATAC peak clusters individually. Each arm of the plot represents an individual metric (center = low, edge = high). Also see Extended Data Fig. 5b.

**Extended Data Fig. 5:**
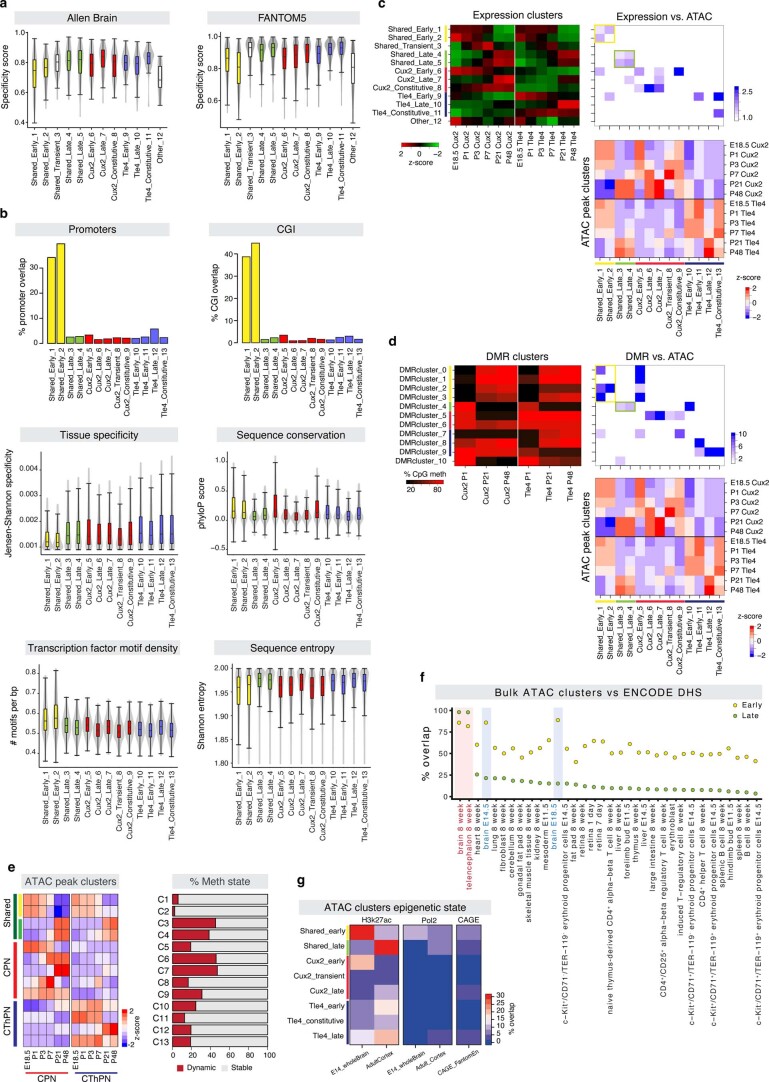
Characterization of RNA-seq and ATAC-seq clusters by cluster. **a**, Expression specificity of genes in each RNA-seq cluster as in Fig. 2c, for each individual cluster. Box and midline: 25th, 50th and 75th percentiles; whiskers: 1.5 interquartile range from box. **b**, Analyses of ATAC peak clusters as in Fig. 2e and H-K, for each individual cluster. Boxplot as in **a**. **c**, Overlap between individual RNA-seq and ATAC clusters. Color scale for top-right panel: odds ratio, Fisher’s exact test, only results with odds ratio >1, BH corrected *P* value ≤0.001, and more than 50 overlapping regions are shown. **d**, Overlap between individual DNAme and ATAC clusters. Color scale for top-right panel: odds ratio, Fisher’s exact test. **e**, Fraction of DMRs in the ATAC clusters that are dynamic versus static over time as in as in Fig. 2f, for each ATAC cluster. **f**, Overlap of early and late shared ATAC clusters with DNAse I hypersensitivity sites in a panel of tissues and cell types from the ENCODE database, by tissue. **g**, Percentage of overlapping ATAC-seq regions for each bulk ATAC-seq cluster for CPN and CThPN (*y* axis) with genomic features derived from E14 whole mouse brain and adult cortex H3K27ac and polymerase 2 ChIP-Seq from ENCODE as well as enhancers defined by CAGE-Seq from the FANTOM5 consortium (*x* axis), excluding all ATAC-Seq regions overlapping with annotated Ensembl promoters.

We then systematically characterized each of these distinct classes of genes and GREs (Fig. 2a). We first assessed the temporal, cell type and tissue specificity of gene expression across the transcriptional clusters (Fig. 2b), for both all genes in each cluster (Fig. 2c) and for transcription factors (TFs) only. We examined expression across a wide range of tissues and cell types, using 77 mouse brain regions and developmental timepoints from the Allen Brain Atlas^41^ and 294 mouse cell types and tissues from the FANTOM5 project^44^ (Fig. 2b,c and Extended Data Fig. 5a). For both all genes and for TFs only, the shared-late genes showed significantly higher temporal and/or cell type specificity in both tissue panels compared to the shared-early genes (*P* ≤ 2.576 × 10^−7^ for all comparisons, one-sided Mann–Whitney test). This suggests that the pan-neuronal genes and TF programs that are active in perinatal postmitotic neurons reflect more broadly used developmental processes compared to those active at later ages.

Interestingly, although some cell-type-specific clusters also showed temporal correlation, early-active and late-active cell-type-specific clusters did not show significant differences in tissue specificity (Fig. 2b,c), indicating that the pan-neuronal and the neuron-type-specific gene programs use different regulatory logic during neuron maturation.

To investigate the relationship between gene expression and ATAC peak cluster dynamics, we calculated the overlap between genes in the transcriptomic clusters and genes putatively regulated by GREs in the ATAC clusters (defined as peaks within 100 kilobases (kb) of the transcriptional start site (TSS)). We found that the shared-early gene expression clusters were most strongly associated with the shared-early ATAC clusters (Benjamini–Hochberg-corrected *P* = 5.748203 × 10^−36^, Fisher’s exact test) and similarly for the shared-late gene expression and ATAC clusters (Extended Data Fig. 5c), indicating that the shared developmental transcriptional and epigenetic programs affect similar sets of genes.

Next, we more finely characterized the putative GREs identified from our ATAC-seq profiles. We found that GREs in the shared-early ATAC clusters were more than ten-fold enriched for annotated promoter regions (within 1 kb upstream or downstream of the TSSs) and CpG islands (CGIs) compared to the shared-late clusters (*P* < 2.2 × 10^−16^, Fisher’s exact test; Fig. 2d,e and Extended Data Fig. 5b). CGIs are strongly associated with TSSs and play an important role in gene regulation^45^. Conversely, members of the shared-late ATAC clusters were largely TSS distal and showed lower frequency of overlap with CGIs and annotated promoters (2.6% and 1.8%, respectively; Fig. 2d,e and Extended Data Fig. 5b), suggesting that they disproportionately function as distal regulatory elements, such as enhancers or insulators. To confirm the enhancer identity of the candidate GREs in our datasets, we examined putative activated enhancers (H3K27ac-positive regions) in mouse brain samples from the mouse ENCODE project^46^ (Extended Data Fig. 5g). We observed enriched overlap of H3K27ac-positive regions from E14.5 whole-brain samples with candidate GREs from the ATAC shared-early clusters and between H3K27ac-positive regions from adult cortex samples with candidate GREs from the ATAC shared-late clusters, supporting the identity as enhancers of the GREs in these clusters.

In agreement with our findings in the transcriptional dataset, the cell-type-specific ATAC clusters did not show a similar transition from promoter to enhancer/insulator usage during neuron maturation. Additionally, the cell-type-specific ATAC clusters showed low overlap with CGIs and annotated promoters regardless of developmental dynamics and, thus, likely act as enhancer regions (Fig. 2d,e and Extended Data Fig. 5b,g). This further supports the suggestion that, during neuronal maturation, neurons use different mechanisms to regulate shared versus cell-type-specific maturation processes.

Given the close relationship between chromatin accessibility and DNAme state, we examined the concordance of these states across cell types and developmental time. We found significant overlap between clusters with similar dynamics, where regions losing open chromatin gained DNAme and vice versa, for shared-early, shared-late and cell-type-specific clusters (Extended Data Figs. 2g,h and 5d). However, the fraction of open chromatin regions exhibiting temporally dynamic DNAme patterns was significantly higher in the shared-late ATAC cluster compared to the shared-early cluster (41.72% versus 4.29%; Fig. 2f and Extended Data Fig. 5e), consistent with the elevated frequency of promoter and CGI regions found in the shared-early clusters and the much higher frequency of distal putative regulatory elements in the shared-late clusters (Fig. 2d,e and Extended Data Fig. 5b,c). Promoter regions are well-known to be much less prone to DNAme changes over development, whereas distal differentially accessible sites frequently coincide with enhancer regions and TF binding sites that exhibit highly dynamic DNAme patterns^47^. This observation suggests that DNA methylation plays a greater role in regulating the shared-late developmental programs by mediating stable silencing of distal putative regulatory elements.

**Extended Data Fig. 6:**
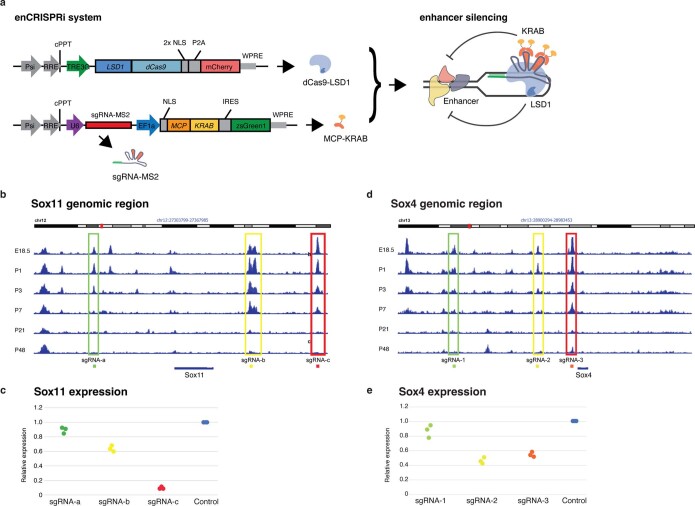
Validation of Sox4 and Sox11 enhancers. **a**, Schematic of enCRISPRi system^48^. **b**, Predicted novel enhancer regions for Sox11 in the mouse bulk ATAC-seq dataset, and location of selected sgRNAs. **c**, Relative Sox11 expression by qPCR after silencing the indicated putative enhancer regions for 5 days (*n* = 3 biological replicates, each performed in duplicate, normalized to an irrelevant control sgRNA). **d**, Predicted novel enhancer regions for Sox4 in the mouse bulk ATAC-seq dataset, and location of selected sgRNAs. **e**, Relative Sox4 expression after silencing the indicated putative enhancer regions for 5 days.

To confirm the biological activity of the predicted GREs, we selected three open chromatin regions each for the *Sox4* and *Sox11* genes and silenced them using the enCRISPRi system^48^ in an in vitro differentiated neuroectodermal cell line (NE-4C; Methods). Inactivation of four of the six predicted GREs resulted in downregulation of their respective gene, indicating that these genomic regions have properties of enhancers and are able to regulate gene expression (Extended Data Fig. 6).

Previous work has suggested that cell-type-specific accessible chromatin sites are preferentially localized in putative enhancer regions compared to promoter regions^4,49–51^. We, therefore, evaluated the tissue specificity of early-active versus late-active GREs across a panel of DNase hypersensitivity sites in 35 adult and embryonic mouse primary tissues and cell types from the ENCODE database^46^. GREs in shared-late developmental epigenetic clusters were, on average, found in an open state in significantly fewer tissues than GREs in shared-early developmental clusters (ATAC clusters early versus late: *P* < 2.2 × 10^−16^, Mann–Whitney test; Fig. 2g and Extended Data Fig. 5f). Notably, late-active ATAC clusters were highly enriched for chromatin regions that are accessible in adult (8-week) mouse brain tissues but not for those open in embryonic brain, adult cerebellum, adult and perinatal retina or any non-central nervous system tissue. In contrast, GREs in early-active ATAC clusters showed high overlap with adult and embryonic brain but also showed broad associations with open chromatin regions in many other cell types and tissues (Fig. 2g and Extended Data Fig. 5f). The ratio of accessible sites suggests that the shared-late active regions are highly enriched for GREs that are specific to the adult brain, consistent with the enrichment of neuron-specific Gene Ontology (GO) processes in these clusters (Supplementary Table 4).

We then compared the average tissue specificity of accessible sites in the shared-early and shared-late ATAC clusters across a single-cell ATAC-seq (scATAC-seq) panel of 85 mouse primary tissues and cell types^17^ (Methods). The shared-early active sites showed lower average specificity (*P* < 2.2 × 10^−16^, Mann–Whitney test; Fig. 2h), again indicating that these sites are more widely used across tissues.

**Extended Data Fig. 7:**
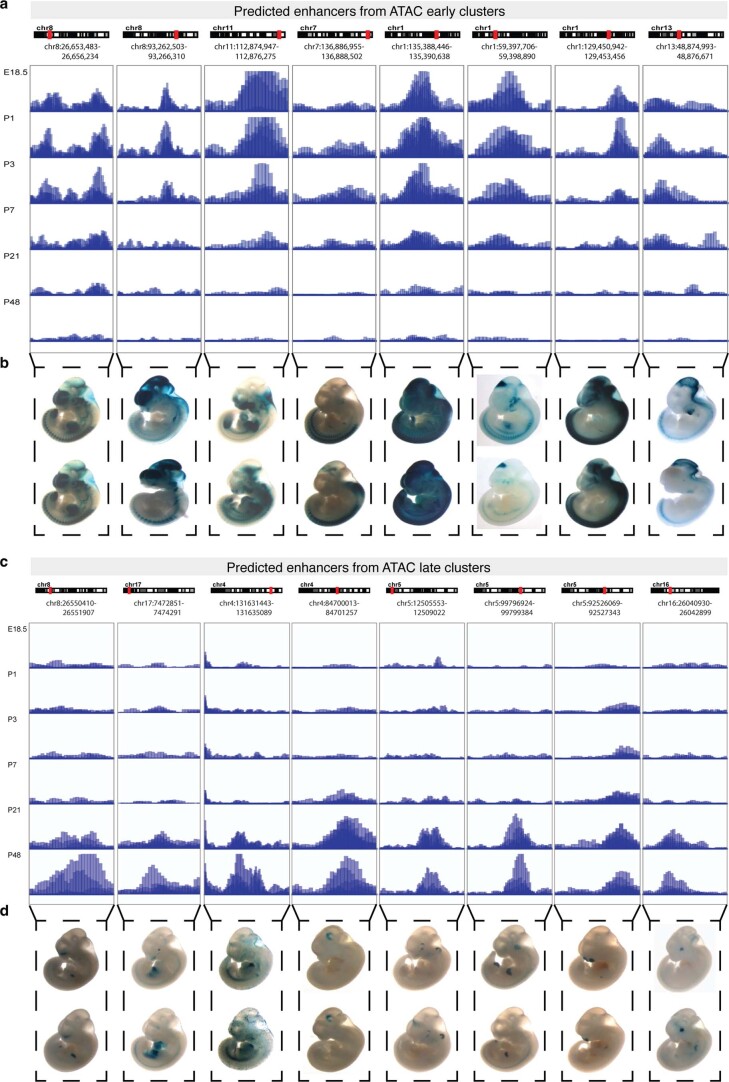
Validating specificity of shared-early and shared-late accessible chromatin sites in the VISTA enhancer dataset. **a**, Example enhancer regions in the VISTA database that span ATAC peaks in the shared-early ATAC cluster, overlaid with ATAC peak accessibility in our data at each age. Top row shows the position of the region on the chromosome. **b**, Expression patterns driven by that DNA segment in E11.5 mouse embryos, from the VISTA dataset. Two examples are shown for each. **c**, Example enhancer regions in the VISTA database that span ATAC peaks in the shared-late ATAC cluster, overlaid with ATAC peak accessibility in our data at each age. Top row shows the position of the region on the chromosome. **d**, Expression patterns driven by that DNA segment in E11.5 mouse embryos, from the VISTA dataset. Two examples are shown for each.

To validate that the shared-early and shared-late accessible chromatin sites from our ATAC-seq data confer different degrees of restriction in expression, we examined the activities of these sites in the VISTA enhancer dataset, in which enhancer activity is visualized by a LacZ reporter assay driven by non-coding DNA fragments in transgenic mice^52^. We examined 16 representative chromatin sites selected from our ATAC-seq data in E11.5 mouse embryos in the VISTA dataset. We found that the early ATAC-seq open regions predominantly drove broad LacZ expression in multiple tissues throughout the organism, whereas the late ATAC-seq open regions drove more selective expression, with LacZ signal visible in fewer tissues (Extended Data Fig. 7), consistent with our bioinformatic analysis (Fig. 2h).

Previous studies of DNA methylation have reported that sequence conservation varies for DMRs characteristic of different tissue types^53^ and that DMRs specific to excitatory cortical neurons are less conserved than those specific to interneurons^16^. Given these observations and the more ubiquitous activity of genes and GREs in the shared-early developmental clusters, we hypothesized that these regions might be under different degrees of evolutionary constraint. Quantifying sequence conservation across placental mammals of the ATAC peak regions in the shared developmentally regulated ATAC clusters found significantly higher conservation of shared-early elements (*P* < 2.2 × 10^−16^, Mann-Whitney test; Fig. 2i and Extended Data Fig. 5b). Shared-early active GREs also showed a higher density of TF binding motifs (*P* < 2.2 × 10^−16^, Mann–Whitney test; Fig. 2j) and lower sequence entropy (a metric of sequence constraint; *P* < 2.2 × 10^−16^, Mann–Whitney test; Fig. 2k), both broadly associated with increased CpG density. Interestingly, across all of these metrics, the neuron subtype-specific gene and GRE clusters largely resembled the shared-late developmental clusters (Fig. 2b–e,h–i and Extended Data Fig. 5b).

Together, these findings (summarized in Fig. 2l) indicate that the shared-early GREs use more widely shared regulatory mechanisms that are consequently under greater evolutionary constraint. In contrast, the shared-late GREs, which have more restricted tissue usage, may be more amenable to variation and may employ more species-specific or evolutionarily recent mechanisms. Notably, we did not find a comparable difference between the cell-type-specific early and late gene and GRE clusters, suggesting that this change in regulatory mechanisms applies specifically to genes and GREs involved in processes shared by all neurons.

### Temporal divergence in global regulatory strategies is a conserved principle of cortical neuron maturation across species

Next, we sought to investigate whether these regulatory principles are common to all neuronal classes in the cortex and, furthermore, whether cortical neurons in other species follow the same principles. Marmosets (*Callithrix jacchus*) are an attractive non-human primate model for neurobiology, offering more human-like brain anatomy and circuitry, cognitive capacities and behavioral repertoires^54,55^. We performed single-cell (mouse) or single-nucleus (marmoset) RNA sequencing (scRNA-seq and snRNA-seq) and single-cell ATAC sequencing (scATAC-seq) on unfractionated cortical tissue from these two species across early and late stages of postnatal development. For mice, we profiled combined somatosensory and motor cortex at three selected ages: P1, when early active gene and GRE clusters predominate; P7, an intermediate stage; and P21, when late active gene and GRE clusters predominate. For marmoset, we profiled somatosensory cortex at neonatal (P1 to P2) and adult ages (2 years (Y2) for scRNA-seq and Y7 to Y8 for scATAC-seq, based on limited tissue availability for this species). We expect that the Y2 and Y7/8 marmoset samples represent comparable adult neurons, given the 12–16-year life span of this species^56^.

**Fig. 3:**
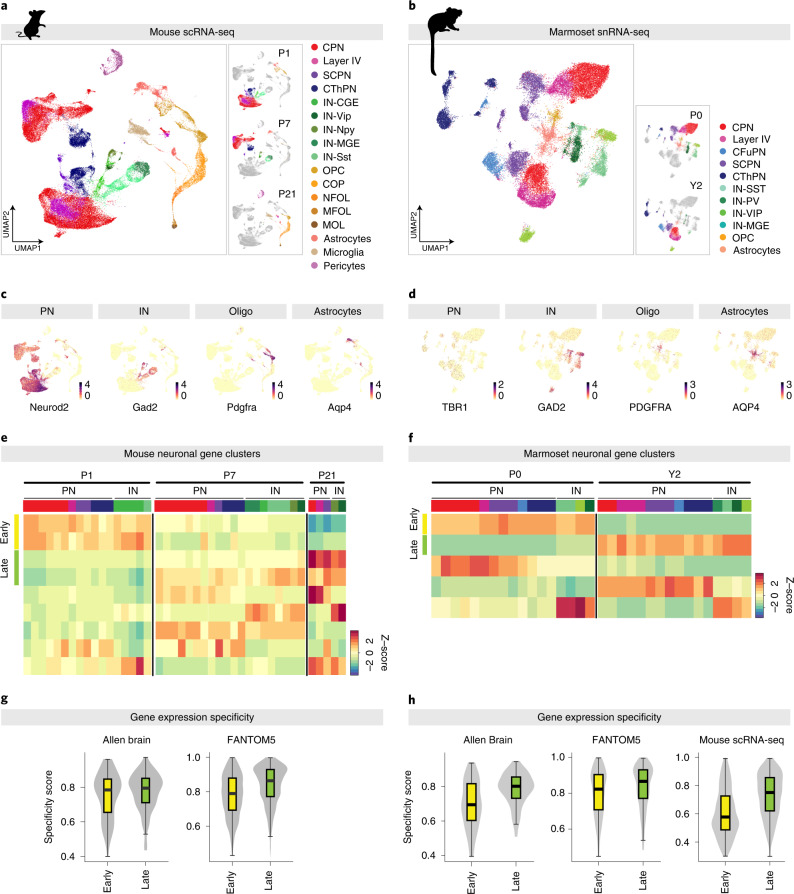
scRNA-seq demonstrates a developmental shift in specificity of shared gene expression programs across multiple neuronal subclasses in both mouse and marmoset. **a**, UMAP representation of gene expression profiles from 60,989 single cells from mouse cortex at P1, P7 and P21, color-coded by major cell type. Left: UMAP plots showing cell distribution by age. **b**, UMAP representation of 36,592 single nuclei from marmoset cortex at P0 and Y2, color-coded by major cell type. Left: UMAP plots showing cell distribution by age. **c**, Representative marker genes for major cell types in the mouse data. Also see Supplementary Fig. 6a,c. **d**, Representative marker genes for major cell types in the marmoset data. Also see Supplementary Fig. 8a,c. **e**, Developmental dynamics of clusters of differentially expressed genes across the mouse excitatory and inhibitory neuronal populations (cell type indicated by color-coded bar at top, corresponding to colors in **a**). **f**, Developmental dynamics of clusters of differentially expressed genes across the marmoset excitatory and inhibitory neuronal populations (cell type indicated by color-coded bar at top, corresponding to colors in **b**). **g**, Mouse gene expression specificity in the shared-early and shared-late gene clusters, within the mouse brain (from expression data from the Allen Brain Atlas; left), and across 397 mouse cell types (from the FANTOM5 project; right). Higher values indicate more specific expression. Box and midline: 25th, 50th and 75th percentiles; whiskers: 1.5× interquartile range from box. **h**, Marmoset gene expression specificity in the shared-early and shared-late gene clusters, within the mouse brain (from expression data from the Allen Brain Atlas; left), across 397 mouse cell types (from the FANTOM5 project; center) and across cell populations from our mouse single-cell dataset (right). Higher values indicate more specific expression. Box plot as in **g**.

We first examined programs of gene expression across the two species. After quality control and filtering, the final dataset contained a total of 60,989 mouse and 36,592 marmoset cells across the different timepoints (Fig. 3a,b and Supplementary Figs. 5–8). We performed cell clustering and assigned cell identity based on expression of known canonical marker genes (Fig. 3a–d and Supplementary Figs. 6 and 8). This identified major neuronal and glial cell populations, including different populations of pyramidal neurons (CPNs, subcerebral projection neurons (SCPNs) and CThPNs) and interneurons, astrocytes and oligodendrocyte-lineage populations, which expressed classical cell type marker genes, including *Neurod2* and *Tbr1* (glutamatergic neurons), *Gad2* (GABAergic interneurons), *Pdgfra* (oligodendrocyte lineage) and *Aqp4* (astrocytes) (Fig. 3c,d). For both mice and marmoset, clusters showed separation by both age and cell type, with projection neuron populations being predominantly separated by age (Fig. 3a,b and Supplementary Figs. 5c and 7c), consistent with our findings that most changes in the mouse bulk sequencing datasets were developmentally related rather than neuron subtype-specific (Fig. 1).

We then identified shared developmental and cell-type-specific gene clusters using unsupervised k-means clustering (Methods), following the same strategy used for the bulk datasets. For each species, we selected all pyramidal neuron and interneuron populations, identified genes that showed differential expression across the sc/snRNA-seq datasets and clustered them by their expression pattern across age and cell type (Fig. 3e,f). For mice, we identified two shared-early and two shared-late developmentally regulated pan-neuronal gene clusters (that is, shared across all pyramidal and interneuron populations), and, for marmoset, we identified one early and one late pan-neuronal gene cluster (Fig. 3e,f).

**Extended Data Fig. 8:**
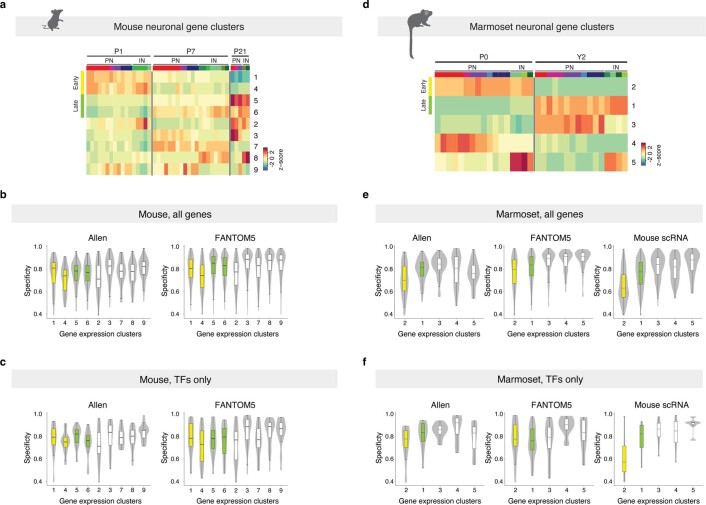
Gene cluster specificity. **a**, Mouse neuronal gene clusters, repeated from Fig. 3 for ease of reference. **b**, Cell-type specificity of all genes in the Allen (left) and FANTOM5 (right) datasets, for each gene cluster. Box and midline: 25, 50 and 75 percentiles; whiskers: 1.5 interquartile range from box. **c**, Cell-type specificity of transcription factors (TFs) in the Allen (left) and FANTOM5 (right) datasets, for each gene cluster. Boxplot as in **b**. **d**, Marmoset neuronal gene clusters, repeated from Fig. 3 for ease of reference. **e**, Cell-type specificity of all genes in the Allen (left) and FANTOM5 (right) datasets, and in our mouse scRNA-seq dataset for each gene cluster. Boxplot as in **b**. **f**, Cell-type specificity of transcription factors in the Allen (left) and FANTOM5 (center) datasets, and in our mouse scRNA-seq dataset (right), for each gene cluster. Boxplot as in **b**.

Next, we analyzed tissue specificity and cell type specificity for TFs in these clusters as we did for the bulk RNA-seq and found that, for both species, the shared-late developmental clusters showed greater TF specificity across both the Allen (brain) and FANTOM5 (tissue) datasets than the shared-early clusters (Fig. 3g–h and Extended Data Fig. 8b,c,e,f). In addition, the marmoset shared-late gene cluster showed greater cell type specificity across our mouse scRNA-seq dataset compared to the shared-early clusters (Fig. 3h). Notably, although the pyramidal and interneuron cell types contained multiple subclusters (both separating different subclasses and further dividing some subclasses), we did not observe striking differences within the subclusters of individual types, indicating that heterogeneity within neuron populations does not explain the gene clustering results. Collectively, these data indicate that the shift in global regulatory principles observed in CPN and CThPN applies to all cortical neuron subtypes and is conserved in the non-human primate cortex.

**Fig. 4:**
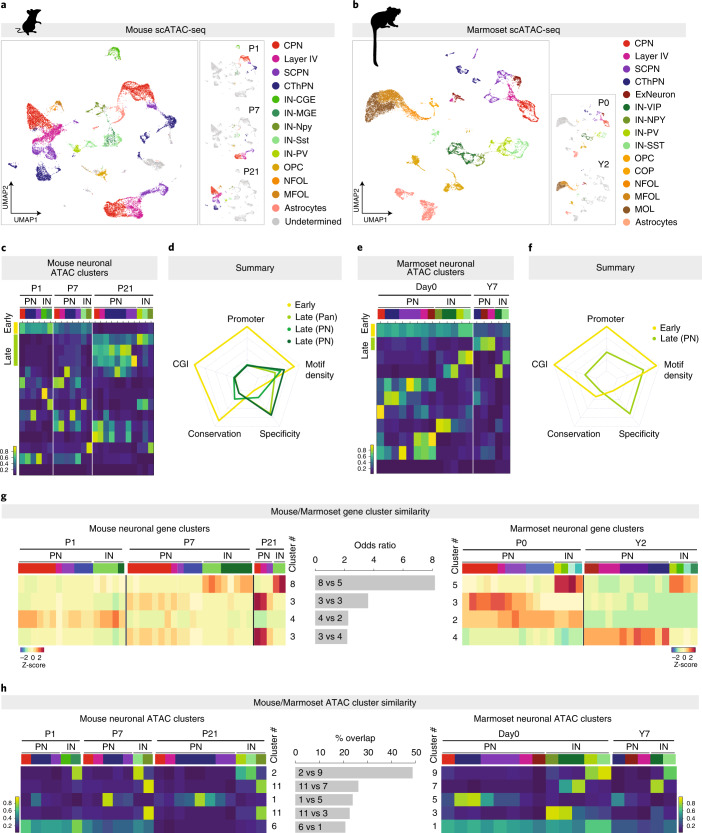
Developmental shift in gene regulatory principles is common to multiple neuronal subclasses and is conserved across mouse and marmoset. **a**, UMAP representation of ATAC chromatin accessibility profiles from 19,145 single cells from mouse cortex at P1, P7 and P21, color-coded by major cell type. Left: UMAP plots with cell distribution by age. **b**, UMAP representation of ATAC chromatin accessibility profiles from 15,919 single cells from marmoset cortex at P0 and Y2, color-coded by major cell type. Left: UMAP plots with cell distribution by age. **c**, Developmental dynamics of clusters of differentially accessible ATAC peaks across the mouse excitatory and inhibitory neuronal populations (cell type indicated by color-coded bar at top, corresponding to colors in **a**). Color scale: average peak normalized read count. **d**, Summary of CGI overlap, promoter overlap, TF motif density, tissue specificity and sequence conservation for each of the mouse shared developmentally regulated clusters, as in Fig. 2k. Also see Extended Data Fig. 9a–c. **e**, Developmental dynamics of clusters of differentially accessible ATAC peaks across the marmoset excitatory and inhibitory neuronal populations (cell type indicated by color-coded bar at top, corresponding to colors in **b**). Color scale: average peak normalized read count. **f**, Summary of CGI overlap, promoter overlap, TF motif density, tissue specificity and sequence conservation for each of the marmoset shared developmentally regulated clusters. Also see Extended Data Fig. 9d–f. **g**, Overlap between genes in the mouse and marmoset single-cell gene expression clusters, showing the four most similar pairs. Also see Extended Data Fig. 10b. **h**, Overlap between accessible regions in the mouse and marmoset single-cell ATAC chromatin accessibility clusters, showing the five most similar pairs. Also see Extended Data Fig. 10d. Color scale: average peak normalized read count.

To examine regulatory characteristics of GREs across cell types and species, we performed scATAC-seq at neonatal and juvenile/adult ages for both species (Fig. 4a,b and Methods). After quality control and filtering, the final dataset included 19,145 mouse and 15,919 marmoset cells (Fig. 4a,b and Supplementary Figs. 9 and 10). Cell type identities were assigned to cell clusters based on inferred expression of the same panel of known marker genes used in the sc/snRNA-seq analyses (Supplementary Fig. 11). Similarly to the scRNA-seq data, neuron populations were predominantly separated by age in both species (Supplementary Figs. 9b and 10b).

**Extended Data Fig. 9:**
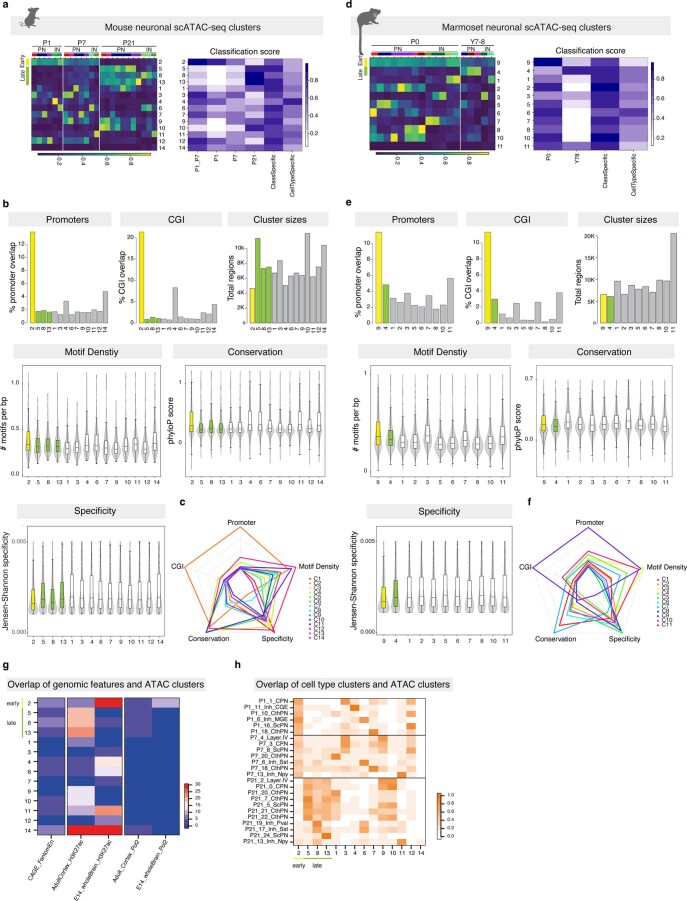
Specificity analyses of mouse and marmoset scATAC peak clusters. **a**, Mouse ATAC peak clusters, reproduced from Fig. 4 for ease of reference (left), and correlation with age versus cell type for each cluster (right). **b**, Mouse ATAC cluster characteristics as in Fig. 4, for each individual cluster, and number of regions per cluster (cluster sizes). Box and midline: 25th, 50th and 75th percentiles; whiskers: 1.5 interquartile range from box. **c**, Summary of characteristics for each cluster in mouse. Each arm of the plot represents an individual metric (center = low, edge = high). Boxplots as in **b**. **d**, Marmoset ATAC peak clusters, reproduced from Fig. 4 for ease of reference (left), and correlation with age versus cell type for each cluster (right). **e**, Marmoset ATAC cluster characteristics as in Fig. 4, for each individual cluster, and number of regions per cluster (Cluster sizes). Boxplots as in **b**. **f**, Summary of characteristics for each cluster in marmoset. Each arm of the plot represents an individual metric (center = low, edge = high). **g**, Percentage of overlapping ATAC-seq regions for scATAC-seq based clusters for all neuronal cell types (*y* axis) with genomic features derived from E14.5 whole mouse brain and adult mouse cortex H3K27ac and polymerase 2 ChIP-Seq from ENCODE as well as enhancers defined by CAGE-Seq from the FANTOM5 consortium (*x* axis), excluding all ATAC-seq regions overlapping with annotated ensemble promoters. **h**, Previously defined average open chromatin signal for neuronal mouse scATAC clusters (*x* axis) shown in a across all neuronal cell types and developmental stages assessed (*y* axis).

We then collapsed differentially accessible ATAC peak regions by cell cluster and clustered these pseudo-bulk profiles. For mice, we identified one pan-neuronal early-peak cluster and one pan-neuronal late-peak cluster, as well as two late-peak clusters shared across pyramidal neuron populations but not interneurons (Fig. 4c). To confirm the biological relevance of these regions, we compared them to histone acetylation chromatin immunoprecipitation followed by sequencing (ChIP-seq) enrichment data from the ENCODE project^46^. The mouse shared-early and shared-late clusters showed high overlap with H3K27ac-enriched regions from embryonic mouse whole brain and adult mouse cortex, respectively, supporting their status as active enhancer elements (Extended Data Fig. 9g,h).

**Extended Data Fig. 10:**
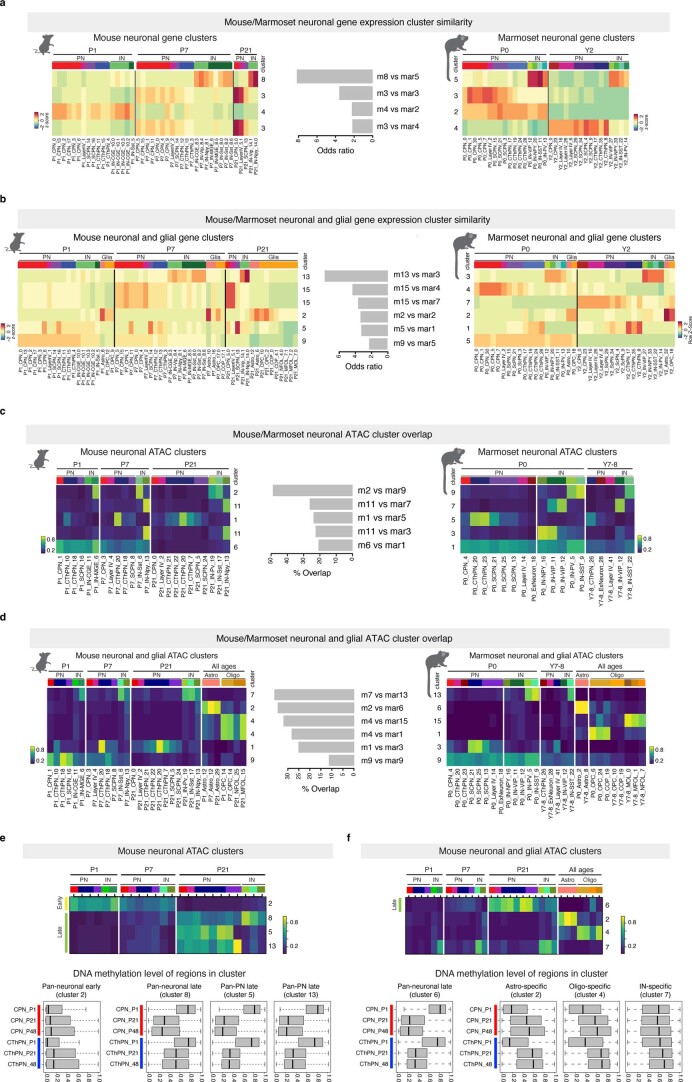
Correlation of mouse versus marmoset gene and ATAC clusters. **a**, Overlap between mouse and marmoset gene clusters for neuronal cell types only (as presented in text), showing the top 4 pairs. **b**, Overlap between mouse and marmoset gene clusters for neuronal and glial cell types taken together (for comparison as a control), showing the top 6 pairs. **c**, Overlap between mouse and marmoset ATAC clusters for neuronal cell types only, showing the top 5 pairs. Color scale: average peak normalized read count. **d**, Overlap between mouse and marmoset ATAC clusters for neuronal and glial cell types taken together, showing the top 4 pairs. Color scale: average peak normalized read count. **e**, Methylation of ATAC peaks in each mouse shared-developmental neuronal ATAC cluster in the mouse bulk DNAme dataset in Fig. 1. Box and midline: 25th, 50th and 75th percentiles; whiskers: 1.5 interquartile range from box. Color scale for ATAC: average peak normalized read count. **f**, Methylation of ATAC peaks in each mouse shared-developmental neuronal and glial ATAC cluster, determined from the mouse bulk WGBS dataset presented in Fig. 1. Boxplots as in **e**. Color scale for ATAC: average peak normalized read count.

Notably, the dynamics of the scATAC peak clusters were correlated with DNAme dynamics (from the mouse bulk dataset): early pan-neuronal scATAC peaks remained mostly unmethylated, whereas late pan-neuronal peaks lost methylation over developmental time (Extended Data Fig. 10e). Interestingly, glia-specific open chromatin regions (such as clusters 2 and 4) showed relatively low DNAme levels in P1 CPNs and CThPNs and only gained DNAme in these neurons during maturation (Extended Data Fig. 10f), consistent with the progressive silencing of alternative lineage programs by DNAme^57^. Interneuron-specific open chromatin regions (cluster 7), however, showed consistently high DNAme levels in CPNs and CThPNs across all ages, suggesting that these programs may need to be silenced at an earlier stage of pyramidal neuron development.

For marmoset, we likewise identified one pan-neuronal early-peak cluster and one late-peak cluster shared across pyramidal neurons but not interneurons. However, we did not identify a pan-neuronal late-peak cluster (Fig. 4e). This may suggest that, in marmoset, adult regulatory programs may diverge more between interneurons and projection neurons than they do in mice, which may reflect greater neuronal specialization in more evolutionarily advanced cortices.

We repeated the ATAC peak characterization previously performed on the mouse Cux2-CPN/Tle4-CThPN bulk sequencing dataset on the scATAC-seq datasets. Consistent with the bulk data, we found that, for both species, the pan-neuronal/pan-projection neuron (pan-PN) early active peak clusters showed greater enrichment for promoters and CGI; increased density of binding motifs (mouse not significant, marmoset early versus late: *P* < 2.2 × 10^−16^); lower tissue specificity (mouse early versus pan-neuronal late: *P* = 0.04574, mouse early versus pan-PN late: *P* < 2.2 × 10^−16^, marmoset early versus late: *P* = 2.282 × 10^−8^); and higher sequence conservation (mouse early versus pan-neuronal late: *P* < 2.2 × 10^−16^, mouse early versus pan-PN late: *P* < 2.2 × 10^−16^, marmoset early versus late: *P* = 0.0008774) (Fig. 4d,f and Extended Data Fig. 9b,c,e,f). Similarly to the sc/snRNA-seq data, we did not observe significant differences between more finely subclustered neuronal subtypes.

Together, these data show that the transition in pan-neuronal developmental programs between broadly used, more highly conserved regulatory elements at earlier stages of postmitotic development versus more cell-type-specific and tissue-specific regulatory elements at later stages holds true across different neuronal classes and is conserved in the non-human primate cortex. The data points at these regulatory principles as broadly generalized properties of neuronal maturation across species.

To compare the similarity of the shared developmental programs between species, we examined the overlap between mouse and marmoset gene and ATAC peak clusters (Fig. 4g,h). We found that the shared-early gene clusters were among the most highly similar pairs of clusters between species; similarly, the shared-early ATAC peak clusters were among the most similar pairs between species (Fig. 4g,h). These findings indicate that general programs of early pan-neuronal development are more frequently shared between species compared to later pan-neuronal programs, which show greater species specificity. This observation is consistent with the broader sequence-level conservation found in shared-early clusters identified from both the bulk pyramidal neuron and scATAC-seq datasets. Interestingly, interneuron-specific clusters were the most highly similar between species for both the gene and ATAC-seq datasets, suggesting that interneuron-specific developmental programs are more highly conserved (Fig. 4g,h).

Lastly, we also compared the mouse bulk ATAC-seq and snATAC-seq clusters to an available snATAC-seq dataset of human fetal cerebrum^58^. This analysis revealed that the mouse shared-early clusters had significant overlap with the human fetal open chromatin regions (Supplementary Fig. 12), suggesting evolutionary conservation of chromatin dynamics at these sites.

In sum, our analysis uncovered a temporal shift in regulatory principles—across multiple modalities and between generalized programs of early (perinatal) and late (juvenile/adult) postmitotic neuronal development—that is found across neuronal subtypes and evolutionarily conserved in rodents and non-human primates.

## Discussion

The neocortex contains a great diversity of neuronal classes that are born during embryogenesis but undergo substantial postnatal maturation to acquire their adult features. Here we have defined a global outline of the regulatory principles underlying key steps of late-embryonic and postnatal development of postmitotic cortical neurons in both rodents and non-human primates. In both species, we uncovered a striking developmental shift between two distinct strategies of epigenomic and transcriptional regulation active at early (perinatal) and late (juvenile/adult) stages of pan-neuronal development. Notably, early and late non-shared (cell-type-specific) programs did not show similar differences. These rules also apply across species, including non-human primates, suggesting that this temporal change in regulatory programs represents a broadly applied, core strategy for cortical neuron development.

Our finding of greater evolutionary conservation of shared, early-active regulatory elements is consistent with previous findings of higher conservation of regulatory elements at earlier stages in bulk forebrain tissue^25^. We now show that this differential conservation applies specifically to shared pan-neuronal regulatory programs but not to cell-type-specific programs active at the same ages and, furthermore, that this strategy is shared by multiple neuron types and conserved across evolution.

A previous study comparing multiple developing and adult human cell types indicated that, whereas programs specific to progenitor stages are often shared between multiple progenitor types, regulatory elements that become active in differentiated cell types are mostly cell type specific^59^. We show here that differentiated, postmitotic neurons nonetheless use a high proportion of GREs with broad tissue and cell type distribution to regulate early pan-neuronal programs. Taken together, these findings suggest that the regulatory programs necessary to produce a baseline cortical neuronal identity are under different developmental and evolutionary constraints from the programs required to confer the distinct, subtype-specific features of each neuronal class. Notably, these results could not have been predicted from analysis of other tissues. Indeed, the greater enhancer usage that we observed in the shared-late GRE clusters contrasts with observations in hematopoetic lineages, where the differentiation and maturation of hematopoietic stem cells into terminal cell types is associated with decreased enhancer usage^32,34,60^.

The data support a conceptual framework in which fundamental events of general, pan-cortical neuron development that occur during perinatal stages, such as establishment of neuronal identity and the acquisition of basic aspects of neuronal architecture, use molecular programs that are shared with other tissues and are, thus, mediated by more generic regulatory programs, requiring a more constrained degree of variation. It is tempting to speculate that this reflects the need for the nervous system to build its basic cell types in a reproducible and invariant manner. In contrast, as neurons transition to phases of neuronal, circuit and synaptic plasticity and function, they employ more specialized transcriptional and epigenetic programs that may allow for more flexibility. The greater variation in cell and circuit behavior at these later stages of cortical maturation may benefit from increased customization, reflected by more rapid species divergence in late developmental regulatory programs, similar to that found in the subtype-specific programs.

## Methods

### Ethics approvals

All animal housing and procedures were conducted in accordance with the US National Institutes of Health *Guide for the Care and Use of Laboratory Animals*. All mouse experiments were approved by the Institutional Animal Care and Use Committee of Harvard University. All marmoset experiments were approved by the Institutional Animal Care and Use Committee of the Massachusetts Institute of Technology.

### Experimental design

No statistical methods were used to predetermine sample sizes, but our sample sizes are similar to those reported in previous publications^61^. For mice, animals were selected according to age, sex and genotype and were randomized within these criteria where possible. For marmosets, sample inclusion was constrained by tissue availability; all tissue was collected from healthy, un-manipulated individuals. Data collection and analysis were not performed blinded to the conditions of the experiments, as the identity of the samples was central to the analysis. The assumptions of the statistical tests used is detailed in the sections for each experimental method, where applicable. No animals or data points were excluded from the analysis.

### Mice

Mice were group-housed in standardized, individually ventilated cages with a 12-hour light/dark cycle, food and water ad libitum, 30–70% humidity and a temperature of 22 °C ± 1 °C. Male and female mice were used for each experiment.

*Cux2*-lineage CPNs were labeled using a *Cux2*-CreERT2 knock-in line^35,62^ (MMRRC 032779-MU). *Tle4*-lineage CThPNs were labeled with a *Tle4*–2A-CreERT2 mouse line^36^ (JAX 036298). The CRE-inducible tdTomato reporter line Ai14 (ref. ^63^) (JAX 007914) was used to detect recombined cells. Animals were maintained on a mixed C57BL/6J background. *Cux2*-CreERT2;Ai14 or *Tle4*-2A-CreERT2;Ai14 double homozygous male mice were crossed with wild-type C57BL/6J females. Cre recombination was induced at E17.5, after the major wave of CPN neurogenesis (E15.5 to E17.5) was complete^64^. 4-Hydroxytamoxifen (4-OHT; Sigma-Aldrich) dissolved in corn oil was administered to pregnant mice at 1 mg of 4-OHT per 10 g of body weight.

For FACS isolation, CRE recombination was induced at E17.5, and the somatosensory cortex and a portion of the motor cortex from transgenic animals was dissected and dissociated at E18.5, P1, P3, P7, P21 and P48. We primarily aimed to analyze somatosensory cortex; however, our dissection strategy was informed by the limited recombination frequency typical of these experiments and the need to collect sufficient tissue to obtain enough labeled cells for bulk RNA-seq, ATAC-seq and WGBS. Tissue dissociation was performed as described^37^, and td-Tomato^+^ cortical pyramidal neurons were isolated by FACS. In brief, cortex was enzymatically digested at 37 °C for 30 min with 10 U ml^−1^ of papain (Worthington Biochemical, LS003126) in dissociation medium (20 mM glucose, 0.8 mM kynurenic acid (Sigma-Aldrich, K3375), 0.05 mM DL-2-amino-5-phosphonopentanoic acid (APV; Sigma-Aldrich, A5282), 50 μl ml^−1^ of penicillin–streptomycin solution (Gibco, 15140122), 0.09 M Na_2_SO_4_, 0.03 M K_2_SO_4_ and 0.014 M MgCl_2_) supplemented with 0.016 μg μl^−1^ of L-cysteine HCl (Sigma-Aldrich, C7477). For P21 and P48 animals, papain concentration was increased to 20 U ml^−1^. Papain digestion was stopped with room temperature dissociation medium supplemented with 10 mg ml^−1^ each of ovomucoid protease inhibitor and BSA (Worthington Biochemical, LK003182), and tissue was mechanically dissociated by gentle trituration in ice-cold Opti-MEM (Gibco, 31985070) supplemented with 20 mM glucose, 0.4 mM kynurenic acid and 0.025 mM APV. We performed RNA-seq and ATAC-seq at E18.5, P1, P3, P7, P21 and P48 and WGBS at P1, P21 and P48. Each library represents a pool of tissue from ten animals (five male and five female) from two litters. Two biological replicates were performed for each age and neuron type for each assay.

### Marmoset

Marmoset tissue was obtained from the laboratory of Guoping Feng at the Massachusetts Institute of Technology. For tissue collection, adult marmosets were deeply sedated with ketamine (20–40 mg kg^−1^, intramuscular) and/or alfaxalone (5–10 mg kg^−1^, intramuscular), followed by intravenous injection of sodium pentobarbital (10–30 mg kg^−1^). Because venous access was not possible in neonates, infant marmosets were sedated with intraperitoneal injection of sodium pentobarbital (10–30 mg kg^−1^). When pedal withdrawal reflex was eliminated and/or respiratory rate was diminished, animals were transcardially perfused with ice-cold PBS or sucrose-HEPES buffer. Whole brains were rapidly extracted into fresh buffer on ice. A series of 2-mm coronal blocking cuts were rapidly made using a custom-designed marmoset brain matrix. Slabs were transferred to a dish with ice-cold buffer, and regions of interest were dissected using a marmoset atlas as reference. Samples of somatosensory cortex were flash-frozen in RNAlater (Invitrogen) or immediately processed for cell dissociation.

### Evaluation of interneuron representation in FACS-purified Cux2-CreERT2 cells

It was reported that the Cux2-CreERT2 line used here also labels a subset of cortical interneurons^35,65^. To evaluate the effects of this on our analysis, we performed single-cell sequencing of a total of 14,792 cells for the Cux2 CPN and Tle4 CThPN populations at three ages, labeled using the same induction strategy used for our primary analysis (Supplementary Fig. 1), using the same approaches as described below for library preparation and analysis. Of these, 239 cells were positive for expression of the interneuron marker Gad1 (1.61%); 339 cells were positive for the interneuron marker Gad2 (2.29%); and 118 cells were positive for both (0.798%), for a total of <5% cells positive for either marker. We conclude that the effect of interneuron contamination on our analysis was minimal.

### RNA-seq of genetically identified projection neuron populations

Pools of 5,000–10,000 cortical pyramidal neurons were sorted directly into TRIzol-LS buffer (Invitrogen), and RNA was extracted according to the manufacturer’s protocol. Ten nanograms of total RNA was used for library preparation using the SMART-Seq v4 Ultra Low Input RNA Kit (Clontech) and the Nextera XT DNA Library Preparation Kit (Illumina) according to the manufacturer’s protocols. All libraries were sequenced according to the manufacturer’s protocols on the HiSeq 2500 system (Illumina), using 125-bp paired-end reads at a depth of >20 million reads per library.

### ATAC-seq of genetically identified projection neuron populations

Pools of 3,000–5,000 cortical pyramidal neurons were sorted into Opti-MEM media (Life Technologies). Nuclei were extracted and libraries prepared following a previously published protocol^66^. All libraries were sequenced according to the manufacturer’s protocols on the HiSeq 2500 system, using 50-bp single-end reads at a depth of ~50 million reads per library.

### WGBS of genetically identified projection neuron populations

Pools of 5,000–10,000 cortical pyramidal neurons were sorted into PBS. The EZ DNA Methylation-Direct Kit (Zymo Research) was used to perform bisulfite conversion, and libraries were prepared with the EpiGnome Methyl-Seq Kit (Illumina). All libraries were sequenced according to the manufacturer’s protocols on the HiSeq 2500 system, using 125-bp paired-end reads at a depth of >200 million reads per library.

### Comparison to human BrainSpan data

We first identified human orthologs of the genes in the mouse shared-early and shared-late gene clusters using the Ensembl database. We then examined expression of these orthologs in human developing brain transcriptomic data from BrainSpan^43^; 1,129 out of 1,334 genes in the shared-early gene cluster and 1,234 out of 1,446 genes in the shared-late gene cluster had orthologs that were expressed in the human data. We then examined expression of these genes within four brain regions (primary motor cortex, primary somatosensory cortex, amygdaloid complex and striatum) over a time course spanning eight (amygdaloid complex) or 12 (all others) post-conceptional weeks to 40 years. Z-scores of the bulk RPKM values were plotted as heat maps using the ‘pheatmap’ package^67^ version 1.0.12 in R version 3.6.0 (Supplementary Fig. 4).

### Comparison to the VISTA enhancer database

We downloaded all enhancers in the VISTA enhancer dataset^52^ from their website (https://enhancer.lbl.gov/) and compared their genomic coordinates to our ATAC data to identify tested regions that spanned ATAC peaks in our shared-early and shared-late clusters. We selected eight representative regions for each cluster and show the ATAC peak signal over each region in our data, as well as the expression pattern it drives in E11.5 mouse embryos in the VISTA dataset (Extended Data Fig. 7).

### scRNA-seq

#### Mouse

Somatosensory and motor cortex from wild-type animals was dissected and dissociated at P1, P7 and P21. For consistency, we collected the same cortical regions as described above for the bulk profiling of transgenically labeled neurons. Each library was made from tissue pooled from at least eight animals, and a balanced sex ratio was used. Tissue dissociation was performed as described above, and live cells were isolated by FACS sorting as DAPI-negative, Vybrant DyeCycle Ruby (Thermo Fisher Scientific)-positive events. Libraries were prepared using the 10x Genomics Chromium Single Cell 3′ kit v2 according to the manufacturer’s protocol.

### Marmoset

Somatosensory cortex from wild-type animals was dissected and flash-frozen in RNAlater (Invitrogen). Each library was made from tissue from an independent individual. Nuclei were extracted by a previously published protocol^68^, and debris was removed by FACS isolation of DAPI-positive nuclei. In brief, cortical tissue was placed in 1 ml of cold nuclei extraction buffer (0.32 M sucrose, 5 mM CaCl_2_, 3 mM Mg(Ac)_2_, 0.1 mM EDTA, 10 mM Tris-HCl and 0.1% Triton X-100) with 10 μl of protease inhibitor cocktail (Sigma-Aldrich, P8340), 1 μl of 100 mM phenylmethylsulfonyl fluoride (PMSF; Sigma-Aldrich, 78830), 1 μl of 1 M 1,4-dithiothreitol (DTT; Sigma-Aldrich, D9779) and 3 μl of 40 U μl^−1^ mRNase inhibitor (Promega, N2611). Nuclei were liberated by dounce homogenization (Sigma-Aldrich, D9063), using 15 strokes with the loose pestle and 25 strokes with the tight pestle. The crude suspension was filtered through a 40-μm nylon mesh cell strainer (Thermo Fisher Scientific, 22363547), transferred to a 15-ml conical tube and centrifuged at 1,000*g* for 10 minutes. The supernatant was removed, and the cell pellet was gently resuspended in 1 ml of cold nuclei extraction buffer with 10 μl of protease inhibitor cocktail, 1 μl of 100 mM PMSF, 1 μl of 1 M DTT and 1.5 μl of 40 U μl^−1^ RNase inhibitor. The suspension was divided between two 1.5-ml microcentrifuge tubes, and each 500-μl sample was gently mixed with 0.75 ml of 50% iodixanol, for a final concentration of 30% iodixanol. The 50% iodixanol solution was prepared by adding 0.4 ml of diluent (150 mM KCl, 30 mM MgCl_2_ and 120 mM Tris-HCl, pH 7.8) to 2 ml of 60% iodixanol (Sigma-Aldrich, D1556). The samples were then centrifuged at 10,000*g* for 20 minutes, and the pellet was resuspended in PBS with 60 U ml^−1^ of RNase inhibitor for FACS isolation. Nuclei were sequenced using the 10x Genomics Chromium Single Cell 3′ kit v2 according to the manufacturer’s suggested protocol for nuclei.

### scATAC-seq

#### Mouse

Somatosensory and motor cortex from wild-type animals was dissected at P1, P7 and P21. For consistency, we collected the same cortical regions as described above for the bulk profiling of transgenically labeled neurons. Libraries were prepared as described in LaFave et al.^69^, a modification of Cusanovich et al.^70^. In brief, cells were fixed with 0.1% formaldehyde and incubated at room temperature for 5 minutes. The fixation was stopped by adding glycine to the final concentration of 125 mM. The sample was incubated at room temperature for 5 minutes and washed in PBS. The cell concentration was counted, and approximately 1,600–2,000 cells per well were distributed into each well of a 96-well plate. Cells were transposed with 96 uniquely barcoded Tn5 at 37 °C for 30 minutes with shaking at 300 r.p.m. The reaction was stopped by adding 0.5 M EDTA and incubated at 37 °C for 15 minutes. All the cells were then pooled, and MgCl_2_ was added to the pooled sample to quench EDTA. The sample was re-distributed onto another 96-well plate with 20 cells in each well by FACS sorting. Reverse crosslinking buffer and barcode PCR primers were added to each sample. The plate was incubated at 55 °C for 16 hours for reverse crosslinking. Tween 20 was then added to quench SDS before PCR amplification.

The PCR reaction was carried out at the following conditions: 72 °C for 5 minutes (extension), 98 °C for 5 minutes and then thermocycling at 98 °C for 10 seconds, 70 °C for 30 seconds and 72 °C for 1 minute for 12–15 cycles. Libraries were pooled and purified using Qiagen MinElute PCR purification column. The libraries were quantified using KAPA library quantification kit. Libraries were sequenced on the Next-seq platform (Illumina) using a 150-cycle kit (Read 1: 47 cycles, Index 1: 36 cycles, Index 2: 36 cycles, Read 2: 47 cycles).

### Marmoset

Somatosensory and motor cortex from wild-type animals was dissected and dissociated with the Worthington Papain Dissociation System (Worthington Biochemical), and live cells were isolated by FACS sorting as DAPI-negative, Vybrant DyeCycle Ruby (Thermo Fisher Scientific)-positive events. Libraries were prepared using the 10x Genomics Chromium Single Cell ATAC kit.

### Immunohistochemistry

To confirm class specificity of labeling, CRE recombination was induced at E17.5 by tamoxifen administration, and mice were sacrificed at E18.5, P1, P3, P7, P21 and P48 for co-immunolabelling with antibodies against the canonical layer markers CUX1, CTIP2 and SATB2.

Mice were deeply anesthetized with tribromoethanol and perfused transcardially with PBS, followed by 4% paraformaldehyde (PFA) in PBS. Brains were post-fixed in 4% PFA overnight, washed in PBS, embedded in low-melting-point agar and sectioned at 20 µm using a Leica VT1000 S vibrating microtome. Sections were transferred to six-well plates with Netwell Inserts (Corning, 3479), washed twice with PBST (1× PBS with 0.2% Triton X-100) and then incubated in blocking buffer consisting of PBST with 8% (v:v) normal goat serum (Invitrogen, 16210–072) or normal donkey serum (Sigma-Aldrich, D9663). Sections were incubated overnight at 4 °C with primary antibodies diluted in blocking buffer, washed in PBST and then incubated with Alexa Fluor-conjugated secondary antibodies diluted in blocking buffer for 2 hours at room temperature. Finally, sections were washed in PBST and mounted with DAPI Fluoromount-G (Southern Biotech, 0100-20). Primary antibodies and dilutions were as follows: mouse anti-Satb2, 1:50 (Abcam, ab51502); rat anti-Ctip2, 1:100 (Abcam, ab18465); and rabbit anti-Cux1 (CDP M-222), 1:300 (Santa Cruz Biotechnology, sc-13024). Secondary antibodies were: goat anti-rat Alexa Fluor 488 (Thermo Fisher Scientific, A48262), donkey anti-mouse Alexa Fluor 647 (Thermo Fisher Scientific, A-31571) and donkey anti-rabbit Alexa Fluor 647 (Thermo Fisher Scientific, A-31573). All secondary antibodies were used at 1:1,000 dilution. Imaging was performed using a Nikon 90i fluorescence microscope equipped with a Retiga EXi camera (QImaging). Analysis was done with Volocity image analysis software version 4.0.1 (Improvision).

### Enhancer silencing by enCRISPRi

We performed the CRISPRi experiment in the mouse neuroepithelial cell line NE4C (American Type Culture Collection (ATCC)). We selected three regions predicted to be open around each of Sox4 and Sox11 in our mouse bulk ATAC dataset. For each of these predicted enhancer regions, we designed three single guide RNAs (sgRNAs) using the Benchling sgRNA design tool (https://www.benchling.com/crispr/).

NE4C cells were cultured following the supplier’s protocol (ATCC). After transfection with the CRISPRi constructs^48^, neuronal differentiation was induced with retinoic acid. Starting 5–7 days after induction of differentiation, doxycycline was added to the plates to induce the dCas9 expression and silence the candidate regions for 5 days. Cells were then collected, and Sox4 and Sox11 expression was quantified by qPCR. Expression was normalized against cells transfected with an irrelevant control sgRNA. Each experiment included three biological replicates, each with two technical replicates.

sgRNA sequences used:

Sox4:

sgRNA1 AGTTAACTGTTTGAGAAAGATG

sgRNA2 CTAAGGTCTTGAGATAAACAGC

sgRNA3 TTAATATAACATGACAGGCACG

Sox11:

sgRNA1 GTCCAACAGCCAGATCTTATAG

sgRNA2: AGTCCTTGCCCATAGTCCTCAG

sgRNA3: GATTGCCTTGATTCCTAAAACG

### Bioinformatics analysis

#### Data processing

##### Bulk RNA-seq

Raw reads were trimmed using Trimmomatic^71^ version 0.33, removing 8 bp from the 5′ end and 25 bp from the 3′ end. Subsequently, reads were aligned to the Ensembl NCBI37 (mm9) genome build (downloaded from the Illumina iGenomes file collection), using TopHat2 (ref. ^72^) version 2.0.13 with default parameters. Subsequently, differential gene expression analysis and FPKM quantification was performed using Cuffdiff^73^ version 2.2.1 for all pairwise comparisons. Differentially expressed genes were defined as all genes that showed a significant (false discovery rate (FDR) ≤ 0.05) change in gene expression with ≥1.5 log_2_ fold change in at least one comparison and were expressed at levels greater than 10 FPKM in at least one condition within that comparison, resulting in *n* = 4,419 differentially expressed genes across the entire dataset.

Next, genes were clustered using k-means clustering on the log_2_-transformed and Z-scored FPKM values of all differentially expressed genes using 100 random starts. To determine the number of clusters, we used the gap statistics as implemented in the R package cluster^74^ version 2.0.7 in combination with the Tibshirani 2001 method^75^ based on the standard deviation evaluating *k* = 2–20, identifying 12 clusters in total. Finally, we classified each cluster manually based on its expression dynamic into one of five categories: shared developmental clusters (1–5), Cux2 CPN-specific (6–8), Tle4 CThPN-specific (9–11) and other (12), as shown in Fig. 1c.

### Bulk ATAC-seq

ATAC-seq raw reads were aligned to the genome build NCBI37 downloaded from the Illumina iGenomes collection using Bowtie2 (ref. ^76^) with default parameters. Subsequently, aligned reads were filtered for duplicates using MarkDuplicates from the Picard software toolbox version 2.7.1 (http://broadinstitute.github.io/picard/)^77^. Next, we performed peak calling for each sample group using the irreproducible discovery rate (IDR) framework^78^ in combination with the macs2 peak caller (MACS2 version 2.1.1), with two independent biological replicates in each group. All peaks detected at IDR ≤ 0.1 in each group were retained for further analysis. We then performed differential peak enrichment analysis across all pairwise group comparisons using the diffBind package^79,80^ in combination with DESeq2 (ref. ^81^). To that end, we employed the DBA_SCORE_TMM_READS_EFFECTIVE score for normalization and subsequent differential enrichment analysis. We defined all peaks exhibiting significant (FDR ≤ 0.01) differential enrichment above a log_2_ fold change of 1.5 and a minimum enrichment ≥1 trimmed mean of M values (TMM) normalized reads in at least one condition as differential, resulting in *n* = 66,784 differentially enriched ATAC-seq peaks across the entire dataset. Subsequently, we averaged over all replicates for each group and transformed the resulting TMM value to log_2_ space. Next, we conducted k-means clustering on Z-scored and log_2_-transformed TMM values on all differentially enriched regions using 100 random starts. We again used the gap statistics in combination with the Tibshirani SE method, which identified 13 clusters. After inspection of the cluster dynamics, we annotated each cluster as shared developmental (split into shared-early and shared-late), CPN-specific, CThPN-specific or other.

Finally, we associated each differentially active ATAC-seq peak with the closest Ensembl gene TSS using the ChIPpeakAnno package^82^. Selected gene names based on this association are shown in Fig. 1d. We then classified each cluster as developmental, neuron class-specific or other according to its dynamic enrichment patterns (Fig. 1d).

### Bulk WGBS

Raw sequencing reads were trimmed 8 bp from the 5′ end and 40–60 bp from the 3′ end, depending on library quality. Next, reads were aligned to the genome build NCBI37 using bsmap^83^ version 2.9 with parameter settings -v 0.1 -s 16 -q 20 -w 100 -S 1 -u –R. Aligned data were then filtered for PCR duplicates using the MarkDuplicates function implemented in the Picard toolbox. Next, CpG methylation calling was performed on the duplicate filtered data using the mcall function implemented in the MOABS suite^84^ version 1.3.2 with default parameters.

### scATAC

Base calls were converted to FASTQ format using bcl2fastq (Illumina). Raw sequencing reads were trimmed using custom Python scripts to remove adapter sequences. The data were demultiplexed tolerating one mismatched base within barcodes. Mitochondrial, unpaired and low-quality reads were removed using SAMtools^85^ version 1.5 (samtools view -b -q 30 -f 0×2). Duplicate sequences were removed using the Picard toolkit^77^. The reads were aligned to the mm10 or CalJac3 genomes using Bowtie2 (ref. ^76^) version 2.3.2 with maximum fragment length set to 2 kb and all other default settings (bowtie2 -X2000–rg-id).

### Data analysis

#### Identification of DMRs

DMRs were identified using the R package DSS^86^. To that end, we performed all pairwise comparisons across sample groups. For each of these pairwise comparisons, we applied the following three functions from the DSS package to the appropriate biological replicates. First, we used the dmlTest with smoothing=T and smoothing.span=200. Next, we identified differentially methylated CpGs using the callDML function with a threshold of *P* = 0.001. Finally, we identified DMRs using the callDMR function directly using the posterior probability that the methylation difference exceeds a certain value. We set the parameters delta=0.3, p.threshold=0.01, minCG=3 and dis.merge=400; all other parameters were left at their default settings. Next, we merged all DMRs identified across the pairwise comparisons into one DMR set, collapsing DMRs that overlap by at least one base pair into a single DMR. For further downstream analysis and visualization, we employed the methylKit package^87^ version 0.9.5. In particular, we computed the methylation level and coverage of each DMR in each sample, defined as the weighted average of CpG methylation levels weighted by coverage. We then retained only those DMRs that were covered by more than five reads in at least three samples. Next, we averaged the methylation levels of each DMR across replicates and assigned the DMRs to different groups based on their methylation differences between the replicate-averaged, DMR-level methylation values. DMRs that exhibited an absolute methylation difference ≥0.3 between any pair of samples and exceeded a size of 100 bp were defined as differentiation DMRs.

We then performed clustering using k-means, initially identifying 12 clusters that we collapsed upon further inspection into ten distinct clusters. We again annotated each cluster according to its dynamic enrichment patterns (Fig. 1e).

We determined global CpG and non-CpG methylation levels as the fraction of methylated CpGs over the total number of detected CpGs (and correspondingly for non-CpGs) for all ATAC-seq regions overlapping with DMRs and covered by at least ten reads in 80% of the samples using the function in the regionCounts function in the methylKit package. We report the corresponding feature methylation values in Extended Data Fig. 2h.

### MDS analysis

We performed MDS using the cmdscale R function with 1 minus the absolute Person correlation coefficient as metric, reducing the dimensionality to two dimensions. We then computed the distances of individual samples shown in Fig. 1g and corresponding text as the two-dimensional (2D) Euclidean distance. As input, we used the log_2_ + 1 transformed FPKM values of all expressed genes (≥10 FPKM in at least one condition) (RNA-seq), the log_2_ + 1 and quantile-normalized ATAC-seq TMM values (ATAC-seq) and the methylation level of all 1-kb tiles of the mouse genome covered by at least five reads in more than two samples.

### Specificity analysis for bulk RNA-seq clusters

We report specificity analysis for differentially expressed TFs as well as gene expression clusters. These specificity analyses were conducted using three distinct datasets that were processed in the following manner:

FANTOM5: Similarly, we computed the expression specificity of TFs in each expression cluster, all TFs and all genes across 294 mouse cell types and tissues based on CAGE data from the FANTOM5 consortium^44^. To that end, we downloaded the CAGE-tag data for promoter regions from the FANTOM5 cell and tissue collection from http://fantom.gsc.riken.jp/5/. We then collapsed all CAGE-tag peaks for each gene by summing up the tag counts, including only primary cell types. Subsequently, we collapsed replicates for each cell type or tissue by averaging.

Allen Brain in situ^41,88^: The Allen brain data were downloaded from the Allen Brain Atlas Developing Mouse Brain website (https://developingmouse.brain-map.org/). We obtained ISH counts for the developing mouse brain at seven distinct fetal timepoints and 11 different brain substructures. We then intersected the resulting list of genes with the list of TFs/genes in each expression cluster and determined the expression specificity of each TF/gene across the 77 conditions (see below for further details) from this atlas. We then plotted the distribution of specificities for each cluster in Fig. 2c for control purposes.

Mouse single-cell RNA-seq data: Here, we used the log_2_-normalized expression values averaged over all the cells in a particular cell cluster using the AverageExpression() function in Seurat. We then used these pseudo-bulk expression values for each gene across all identified cell types as input for the specificity analysis.

Similarly, we computed the expression specificity of TFs in each expression cluster, all TFs and all genes across 294 mouse cell types and tissues based on CAGE data from the FANTOM5 consortium^44^. To that end, we downloaded the CAGE-tag data for promoter regions from the FANTOM5 cell and tissue collection from http://fantom.gsc.riken.jp/5/. We then collapsed all CAGE-tag peaks for each gene by summing up the tag counts.

We then computed expression specificity for each TF/gene following previous approaches using the tau specificity measure^89^ according to^90^:

$$\tau = \frac{{\mathop {\sum }\nolimits_{i = 1}^n (1 - \widehat {x_i})}}{{n - 1}};\widehat {x_i} = \frac{{x_i}}{{\mathop {{\max }}\limits_{1 \le i \le n} x_i}}$$

with *n* being the number of samples/tissues and x_i_ being the expression of the gene in tissue *i*. We report the result in Fig. 2c.

### Specificity analysis for scATAC clusters

To assess the specificity of each ATAC peak in the bulk or scATAC dataset, we downloaded pre-computed ATAC peak specificity scores computed over more than 80 distinct cell types of an entire mouse using scATAC-seq data from Cusanovich et al.^17^.

We then intersected our peak library with the Cusanovich et al. dataset and report the Cusanovich et al. specificity values for all peaks that overlap with at least one peak in the Cusanovich et al. dataset.

### Bulk ATAC peak overlap with DNAse data

To create a catalog of gene regulatory elements in the mouse genome, we downloaded a set of DNAse HS I peak tracks from the mouse ENCODE consortium^46^ (Supplementary Table 5). Subsequently, we collapsed replicates for each condition and required that each peak was present in at least two replicates. This step resulted in DNAse I tracks for 35 distinct primary mouse cell types and tissues. Next, we merged all DNAse I tracks into a union peak set using the reduce() function in the IRanges R package^91^. Subsequently, we size-standardized the resulting union peak set to 350 bp by extending 175 bp from the center of each peak. Next, we assigned a binary value for each peak in each of the 35 cell types, depending on whether or not the peak was present in the individual cell-type-level peak set. We then used this union peak set and overlapped all ATAC peaks from the mouse bulk ATAC-seq dataset with this library. We report the percent of peaks in each cluster that overlap DNAse I HS sites in each cell type.

### Definition of genomic features

CGIs were defined as previously described^92^ and are listed in Supplementary Table 6. Promoters were defined as all NCBIm37 Ensembl version 67 TSSs, extended by 1 kb upstream and downstream.

### TF binding site density analysis

For each set of regions of interest (DMRs and ATAC peaks), we performed motif detection analysis using FIMO^93^ with a *P* value filter of less than 10^−4^ and a joint motif database comprising the TRANSFAC Professional library (version 2011)^94^ and a set of previously published motifs by Jolma et al.^95^. All genomic regions were size-standardized (if not already) before motif analysis.

### Entropy analysis

To compute the Shannon entropy of the size-standardized ATAC-seq peak sequence, we used the entropy() function in the sequtils^96^ Python package.

### Integrative analysis

To evaluate the concordance of changes in the transcriptome, open chromatin and DNA methylation landscape, we associated each ATAC-peak or DMR with its nearest gene within 100 kb upstream or downstream. Peaks/DMRs without any assigned gene were not considered. Subsequently, we performed a hypergeometric test between all pairs of ATAC-peak/DMR clusters and bulk expression clusters in gene space to assess the significance of overlap. After multiple-testing correction using the Benjamini–Hochberg method^97^, we report the odds ratio of associations significant below a *q* value of 0.001 in Extended Data Fig. 5c,d, capping the odds ratio at 10.

### Phylogenetic conservation analysis

We performed phylogenetic conservation analysis for ATAC-seq cluster groups by computing the average placental mammal phyloP scores^98^ for each region. We then plotted the distribution of these mean scores.

### Signature gene set analysis

First, we associated each of our consensus DMRs with the nearest mouse Ensembl TSS within 100 kb using the ChIPPeakAnno R package^82,99^. Subsequently, we performed gene-set-level analysis and determined the mean methylation level of all DMRs associated with a member gene of each signature gene set using the aforementioned DMR–gene associations. We used signature gene sets for CThPNs, CPNs and SCPNs obtained from the DeCoN database^12^ as well as manually curated gene sets for glial and interneuron cell types from published transcriptomic data^100,101^. We then report the mean methylation level of all DMRs associated with each gene set in Extended Data Fig. 2c for each timepoint and cell type.

### Global methylation level analysis

Global CpG methylation level was defined as the total number of detected methylated Cs in CpG context over the total number of CpGs sequenced. Similarly, we defined the global non-CpG methylation level comprising all other dinucleotide contexts.

### scRNA-seq analysis

Mouse scRNA-seq data were processed using Cell Ranger version 3.0.1 (10x Genomics) using standard parameters and genome assembly GRCm38 downloaded from the 10x Genomics website. After initial alignment and processing by cellranger count, all replicates across all timepoints were aggregated using the cellranger aggr function, downsampling the individual libraries to a similar overall coverage by cell.

All following analyses were conducted in R using the Seurat package (version 2.3.4). We first filtered the dataset to retain only cells with at least 1,000 genes, a mitochondrial read fraction below 10% and not more than 6,000 genes or 15,000 unique molecular identifiers (UMIs).

Subsequently, we initially performed cell clustering and subtype identification separately for each timepoint P1, P7 and P21. For that purpose, we subsetted the data for each timepoint and then applied the following workflow. Expression data were normalized using the LogNormalize method, and variable genes were identified based on the mean/dispersion relation (initially using the following parameters: x.low.cutoff = 0.05, x.high.cutoff = 3, y.cutoff = 0.05).

Next, the data were scaled using the ScaleData function, regressing out percentage of mitochondrial reads and UMI count per cell. We then computed the first 100 principal components (PCs) and examined the resulting elbow plot for variance explained by each PC. Based on that, we selected the first 35–40 PCs for subsequent dimensionality reduction by uniform manifold approximation and projection (UMAP) (min_dist = 0.2 to 0.5) and cluster identification using the Seurat FindClusters (resolution = 0.4 to 0.6, nn.eps = 0.5) function.

We conducted several rounds of principal component analysis (PCA), clustering and UMAP embedding, successively removing clear outlier cell clusters based on the UMAP (four for P1, two for P7 and two for P21).

After cleanup and cluster identification by timepoint, we analyzed all timepoints jointly, assigning the cell original cluster definitions based on the individual timepoint analysis. For that purpose, we used as cutoffs in the variable gene feature analysis: x.low.cutoff = 0.025, x.high.cutoff = 3, y.cutoff = 0.05, 50 PCs, a resolution of 0.9 in the clustering analysis and a min_dist = 0.4 in the UMAP.

Next, we performed cell type identification for each cluster using a set of manually curated marker genes for each cell type, identified from previous literature, the DeCoN database^12^ (for projection neuron subtypes) and published transcriptomic data for glial and interneuron cell types^100,101^. To assign identities, we examined the expression of all marker genes individually using the UMAP. In addition, we computed the AverageExpression for each cluster and examined the pseudo-bulk profiles. In particular, we computed joint cell type scores for each cluster and potential identity by normalizing the average cell type scores for each cluster to the maximum observed score for each cell type separately. Moreover, we performed hierarchical clustering, correlation analysis, PCA and MDS evaluation of the pseudo-bulk profiles to identify outlier clusters and investigate the relationship between cell clusters in more detail. Based on these analyses, we were able to assign identities to most cell clusters. For a few clusters that appeared to have mixed identities (such as a cluster containing multiple subtypes of interneurons), we performed subclustering to refine the identities of these cells. We then collapsed subclusters with the same subtype identity into a single subtype cluster to reduce the complexity for subsequent analysis (final cluster *n* = 32).

### Differential expression analysis

To identify differentially expressed genes between timepoints within distinct cell type classes, we assigned each cell cluster to seven sets of general cell classes that we analyzed separately: excitatory neurons, inhibitory neurons, astrocytes, oligodendrocytes, neurons, glia and all cell types combined.

We then performed differential expression analysis in pairwise fashion between P1 and P7, P1 and P21 and P7 and P21 for each of the aforementioned cell type classes using the FindMarker function in Seurat and the MAST method for differential expression testing.

In this manner, we obtained seven distinct lists of genes differentially expressed across development. Next, we averaged the expression of all cells within one cluster using the AverageExpression function in Seurat. To avoid biases driven by cluster complexity and cell number, we downsampled each cluster to a maximum of 500 cells per cluster before averaging. Next, we performed clustering on Z-score-transformed expression values of each of the seven differentially expressed gene sets separately. For that purpose, we used the clusGap function in the R package cluster^74^ using kmeans with a maximum of 20 clusters and the B parameter set to 60. We then select the final number of clusters based on the Tibshirani 2001 criterion^75^ for the standard deviation as implemented in the cluster package. Finally, we performed k-means clustering with 100 random starts using the identified number of clusters and average over the Z-scored genes in each cluster (Fig. 3 and Extended Data Fig. 8).

Lastly, we performed expression specificity analysis for all gene clusters in a similar manner as for the bulk data, using the Allen Brain in situ dataset, the FANTOM5 and the mouse scRNA-seq low-resolution dataset for comparison for all differentially expressed genes as well as for TFs only.

### Marmoset snRNA-seq analysis

Marmoset snRNA-seq data were processed using Cell Ranger version 2.1.0 and aligned to a pre-mRNA custom build transcriptome of assembly version ASM275486v1.93. All replicates were first processed independently using cellranger count and then aggregated, downsampling all libraries to the same complexity per cell. Subsequently, the data were processed using Seurat following a similar workflow as for the mouse scRNA-seq, retaining only cells with more than 1,000 genes and genes detected in more than ten cells. Initially, variable genes were again identified using the mean/dispersion relation with the following parameters: x.low.cutoff = 0.05, x.high.cutoff = 3, y.cutoff = 0.05. During initial quality control of the five individual marmoset libraries, we observed a separation by experimental batch, where all three libraries (D0 and Y2) from experimental batch 1 and the two libraries from batch 2 (D0 and Y2) grouped together. Given that developmental timepoint and experimental batch are not confounded in our experimental design, we performed batch correction using CCA as implemented in the Seurat package. Here, we discarded all cells where the variance explained by CCA is <2-fold. After correction, a clear separation by timepoint and cell type became apparent, as originally observed when processing each experimental batch separately. We thus proceeded with the CCA-corrected data following the same strategy as for the mouse scRNA-seq data, performing PCA and using 20 dimensions in subsequent analyses, identifying clusters with a resolution of 1.8 and creating a UMAP embedding with a min_dist of 0.3.

Subsequently, data analysis was conducted similarly to the mouse scRNA-seq data, assigning cell types and cell classes, performing differential expression analysis between timepoints within each of seven cell classes, collapsing clusters with similar subtype identity and performing gene clustering within each cell group to identify gene clusters.

### scATAC analysis

#### Read alignment and pre-processing

Base calls were converted to FASTQ format using bcl2fastq (Illumina). Raw sequencing reads were trimmed using custom Python scripts to remove adapter sequences. The reads were aligned to the mm10 or CalJac3 genome using Bowtie2 (ref. ^76^) with maximum fragment length set to 2 kb and all other default settings (bowtie2 -X2000–rg-id). The data were demultiplexed tolerating one mismatched base within barcodes. Mitochondrial, unpaired and low-quality reads were removed using SAMtools^85^ (samtools view -b -q 30 -f 0×2). Duplicate sequences were removed using the Picard toolkit^77^. To counteract differential complexity across the libraries, each library was sampled to a similar fragment depth per cell.

Mouse and marmoset libraries were first downsampled to similar overall complexity in terms of reads per cell across all conditions. Cleaned data were processed with the Scasat^102^ pipeline using macs2 as peak caller with parameters set to -q 0.2–nomodel –nolambda, giving rise to a peak × cell matrix count matrix. For genome size, we used -g mm for mouse and -g 2.1e + 9 for marmoset. We then size-standardized all peaks to 500 bp and recomputed the peak × cell count matrix, considering only reads overlapping the size-reduced peaks.

Only cells with at least 1,000 reads and 300 peaks from the master list overlapping with at least one read, but not more than 15,000 reads and 8,000 peaks, were retained for analysis. In addition, only peaks present in at least 20 cells were retained.

Subsequently, this matrix was processed using R implementing a custom processing pipeline based on the strategy outlined in Cusanovich et al.^17^ and refined by Hill^103^, following the log-latent semantic indexing (log-LSI) workflow. In brief, the count matrix was first binarized and then transformed using the TF-IDF method^70^, log-scaling the results. Next, PCA was performed on the transformed matrix using 50 dimensions, after cell cluster identification based on Seurat’s FindCluster() function with resolution set to 0.5 and UMAP embedding with a min_dist of 0.3. Notably, we split each cluster containing cells from different timepoints into separate clusters for each timepoint and then filtered out all clusters with fewer than 100 cells.

### Cell type identification in scATAC data

To reliably identify distinct cell types, we next computed gene activity scores for all genes based on the presence of ATAC peaks. To that end, we used the R package Cicero^104^, because it implements an approach considering not only peaks located within the promoter/gene body of each gene but also weights the contribution of each peak to the overall gene score based on the correlation of the gene peaks with each other. To that end, we imported the pre-processed data into a cicero atac_cds object providing the cluster ID and UMAP coordinates defined based on the aforementioned LSI analysis. Next, we estimated library size factors and reliable peaks using default parameters, followed by running Cicero’s main function with default parameters to compute the connectivity graph of all peaks. Next, we annotated all peaks with the annotate_cds_by_site function using the transcription start coordinates for all Ensembl genes retrieved from biomaRt, defining the region ±5 kb of each TSS as the promoter region. With this annotation in place, we constructed the raw gene activity matrix using the build_gene_activity_matrix function, followed by normalizing the resulting scores using the function normalize_gene_activities.

Next, we averaged the computed gene scores for each cell cluster and normalized the aggregated score for each gene to the maximum across all pseudo-bulk cell clusters. We then again computed cell type scores for each scATAC cell cluster by averaging a set of known marker genes (the same as used for scRNA-seq) for each cluster and plotting results along with hierarchical clustering, PCA and MDS analysis of the pseudo-bulk gene scores. Based on these analyses, we removed outlier clusters (for example, very low complexity) and assigned a final cell type annotation. Based on this cleaned dataset, we performed differential accessibility analysis, aggregating the signal of all cells in each cluster using Cicero’s aggregate_by_cell_bin function, followed by a negative binomial-based differential accessibility test implemented in the differentialGeneTest function using the cluster ID as a factor to test. In addition to the cell-cluster-based test, we also performed a second test on the timepoint variable to identify specifically those peaks variable across distinct timepoints. We then selected all peaks below a *q* value of 0.5 in either of the two analyses as a dynamic peak set for subsequent analysis. The results are not particularly sensitive to this threshold, as we tested *q* value thresholds between 0.1 and 0.5. However, given the limited power for each peak, we decided to include all peaks with some evidence for differential accessibility in further clustering analysis. We opted to use a 0.5 threshold, as this gave us a similar fraction of variable peaks as the bulk ATAC analysis conducted earlier.

All subsequent analyses were conducted on this dynamic peak set, after excluding all cells of unassigned identity. Next, we normalized the read counts for each cell by the respective size factor and averaged the resulting values across all cells within one cluster, giving rise to pseudo-bulk profiles. Next, we normalized these profiles for each peak across all pseudo-bulk clusters by dividing each row (corresponding to peaks) by the 95th quantile across all cell clusters, capping all values at 1. This gave rise to a normalized accessibility score in the unit interval. These values were then used for clustering analysis, following the same strategy as for the scRNA-seq, again assigning each cell cluster to one of seven categories and then performing k-means with 100 random starts and clustering on the respective subset of cell clusters for all peaks in the dynamic peak set. Again, the cluster number was determined using the clusGap function in the R package cluster and Tibshirani 2001 SE criterion. The resulting peak clusters were then subjected to the same characterization as the peak clusters from the bulk ATAC analysis. For the specificity analysis, the cluster coordinates were lifted from mm10 to mm9 using the USCS liftover tool (http://genome.ucsc.edu/)^105^.

### Defining early, late and cell-type-specific clusters

To assign clusters to a particular activity pattern to individual region clusters, we predefined a set of patterns according to possible dynamics of interest. These included temporal neurons (for example, specific to P1, P7, P21 or P1_P7 or Y0 and Y2), excitatory neurons and inhibitory neurons as well as specific to a particular cell cluster. These patterns were summarized in a prototype binary indicator matrix of dimensions number of cell clusters times number of patterns. For each pattern and cell cluster, the indicator matrix cell was set to 1 if that cell cluster was associated with that pattern—for example, setting the indicator variable for P1_CPN_1 and P1_CPN_3 both to 1 in the P1 pattern definition.

We then computed the cosine similarity between each of these pattern vectors with the normalized accessibility score vector for each region cluster, taking the average of accessibility score for all regions within one region cluster for each cell cluster separately. This analysis gave rise to a similarity measure of each cluster’s dynamic to a predefined set of temporal/cell type dynamics. Based on the maximum observed similarity to a predefined pattern, we then assigned the pattern label to the corresponding cluster.

### Mouse single-cell ATAC peak methylation analysis

Before DNA methylation analysis, we performed a liftover of the size-standardized (500-bp) scATAC peaks from mm10 to mm9 using the UCSC liftover tool. Next, we computed DNAme levels in a similar manner as for the bulk data, using the scATAC peaks instead of the DMR regions as input, considering only regions that were covered by at least five reads in at least eight of the 12 WGBS samples. We report the distribution of methylation levels for each of the scATAC region clusters across CPN and CThPN neurons at P1, P21 and P48.

### Similarity in cell types across ages

To compute the similarities among cell types within each age group and compare them, we first merged single-cell data into pseudo-bulk by computing the average expression for each cell type at each timepoint (using the AverageExpression function from Seurat package version 3.1.0 in R version 3.6.3). We then performed PCA (using the prcomp function included in R as its base package), taking the top ten PCs (ordered by the fraction of total variance explained), to project the data onto the two dimensions using the UMAP algorithm (umap package version 0.2.4.0 in R).

### GO enrichment analysis

We prepared two input gene lists for each organism (shared-early and shared-late in the neuronal cell population) based on our gene module clustering. To query marmoset genes, we first converted them into human (GRCh38.p13) orthologous genes (using the Ensembl database). Enrichment analysis was done using the Panther overrepresentation test available through the Gene Ontology Consortium^106–108^ web interface. Test type was set to Fisher’s exact test, and FDR was computed for each term. Selected GO terms for the shared developmental clusters are presented in Supplementary Table 4.

### Computation of mouse/marmoset gene and open chromatin cluster similarity

To determine overlap of mouse and marmoset gene clusters with each other, mouse Ensembl gene IDs were mapped to marmoset gene IDs using the Ensembl homolog database, and only mappable genes with known homologs were retained for further interspecies analysis. With this mapping in hand, we determined the overlap of all mouse and marmoset gene clusters, determining significance and odds ratio using Fisher’s exact test with the union of all mappable and clustered genes (containing only genes differentially expressed in each dataset) as background set. These results are shown in Fig. 4g.

Open chromatin cluster overlap based on scATAC-seq between mouse and marmoset clusters was determined by first mapping size-standardized (500-bp) clustered marmoset open chromatin peaks to the mouse genome using bnMapper from the bx-python software suite (https://github.com/bxlab/bx-python) and mapping file calJac3ToMm10.over.chain.pkl downloaded from the UCSC genome browser website. Subsequently, the resulting mapped regions were filtered to be at least 100 bp in size and showing a ratio of mapped region size to original region size of less than 1.2. All regions fulfilling these criteria were used for further analysis. We then compared the pairwise overlap in terms of genomic regions for each mouse and marmoset open chromatin cluster and report the percentage of overlap in Fig. 4f.

Similarly, we computed the overlap with open chromatin regions in human fetal cerebrum from ref. ^58^ (downloaded from https://descartes.brotmanbaty.org/) for mouse and marmoset scATAC-based clusters, mapping them to hg19 analogous to the strategy described above. Although this dataset is not restricted to the cortex, at the time of this study comparably large single-cell ATAC datasets for the human fetal cortex were not available.

### Reporting summary

Further information on research design is available in the Nature Research Reporting Summary linked to this article.

## Online content

Any methods, additional references, Nature Research reporting summaries, source data, extended data, supplementary information, acknowledgements, peer review information; details of author contributions and competing interests; and statements of data and code availability are available at 10.1038/s41593-022-01123-4.

## Supplementary information

Supplementary Information Supplementary Figs. 1–12

Reporting Summary

Supplementary Table 1 Dynamic genes in the Cux2/Tle4 bulk dataset. fClustId, final cluster assignment. clustLabel, cluster type description.

Supplementary Table 2 Dynamic ATAC peaks in the Cux2/Tle4 bulk dataset. fClustId, final cluster assignment. clustLabel, cluster type description.

Supplementary Table 3 Dynamic DMRs in the Cux2/Tle4 bulk dataset. fClustId, final cluster assignment. clustLabel, cluster type description.

Supplementary Table 4 GO terms associated with shared developmental Cux2/Tle4 gene expression clusters (tabs 1–5) and with shared developmental Cux2/Tle4 ATAC peak clusters (tabs 6–9). Selected GO terms from the highest-fold enrichment terms for each early developmental and late developmental cluster. Test results of Fisher’s exact test are reported as *P* values, and multiple comparisons were adjusted using Benjamini–Hochberg correction.

Supplementary Table 5 DNAse HS I peak tracks from the mouse ENCODE consortium used in this analysis

Supplementary Table 6 List of CpG islands

## Data Availability

The datasets generated in the current study are available through the Gene Expression Omnibus (GEO SuperSeries GSE204851). The following publicly available datasets were used: *Mus musculus* Ensembl genome build NCBIM37 (mm9); *Callithrix jacchus* (common marmoset) genome assembly version ASM275486v1.93; human developing brain transcriptomic data from BrainSpan^43^ (http://brainspan.org/rnaseq/search/index.html); mouse enhancer data from the VISTA enhancer database^52^ (http://enhancer.lbl.gov/); mouse CAGE-tag data from the FANTOM5 database^44^ (https://fantom.gsc.riken.jp/5/); mouse ISH expression data from the Allen Brain Atlas Developing Mouse Brain database (http://developingmouse.brain-map.org/); mouse single-cell ATAC-seq data from Cusanovich et al.^17^ (GEO: GSE111586); DNAse HS I peak tracks from the mouse ENCODE consortium^46^ (www.encodeproject.org); and human fetal cerebrum single-cell ATAC-seq data from Domcke et al.^58^ (https://descartes.brotmanbaty.org/).

## Code availability

All custom scripts are available from the corresponding authors upon reasonable request.
