# Supplementary material for: Temporally divergent regulatory mechanisms govern neuronal diversification and maturation in the mouse and marmoset neocortex
Source: Nat Neurosci. 2022 Aug 1;25(8):1049–58. doi: 10.1038/s41593-022-01123-4 (PMC9343253; doi:10.1038/s41593-022-01123-4)
Supplement: Supplementary file 1 — Supplementary Figs. 1–12 [file 41593_2022_1123_MOESM1_ESM.pdf]

nature portfolio

https://doi.org/10.1038/s41593-022-01123-4

# Supplementary information

# Temporally divergent regulatory mechanisms govern neuronal diversification and maturation in the mouse and marmoset neocortex

In the format provided by the authors and unedited

**Supplementary Information:**

**Supplementary Figures 1-12**

**Supplementary Table 1.** (separate tab-delimited text file)

Dynamic genes in the Cux2/Tle4 bulk dataset. fClustId, final cluster assignment. clustLabel, cluster type description.

**Supplementary Table 2.** (separate tab-delimited text file)

Dynamic ATAC peaks in the Cux2/Tle4 bulk dataset. fClustId, final cluster assignment. clustLabel, cluster type description.

**Supplementary Table 3.** (separate tab-delimited text file)

Dynamic DMRs in the Cux2/Tle4 bulk dataset. fClustId, final cluster assignment. clustLabel, cluster type description.

**Supplementary Table 4.** (separate Excel file)

GO terms associated with shared-developmental Cux2/Tle4 gene expression clusters (tabs 1-5) and with shared-developmental Cux2/Tle4 ATAC peak clusters (tabs 6-9). Selected GO terms from the highest fold enrichment terms for each early and late developmental cluster. Test results of Fisher's exact test are reported as p-values and multiple comparison adjusted using the Benjamini-Hochberg correction.

**Supplementary Table 5.** (separate tab-delimited text file)

DNAse HS I peak tracks from the mouse ENCODE consortium used in this analysis.

**Supplementary Table 6.** (separate tab-delimited text file)

List of CpG islands.

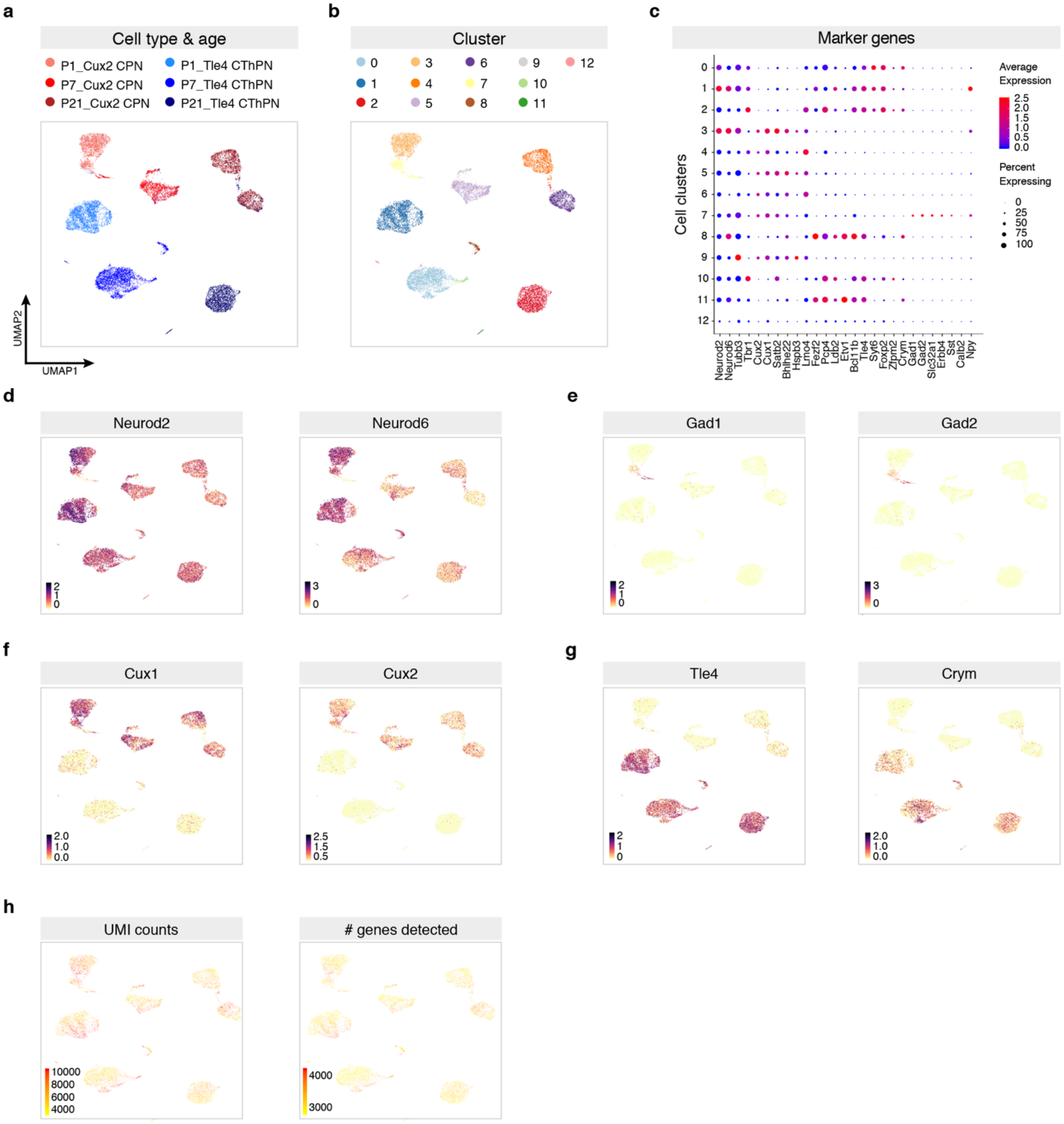


**Supplementary Fig. 1. Verification of subtype identity of genetically-labelled mouse PN subtypes by single-cell RNA-seq of FACS-purified Cux2 CPN and Tle4 CThPN. a**, UMAP showing distribution of cells by line and age. **b**, UMAP showing cell clusters after k-means clustering. **c**, Average expression of selected marker genes in each cluster. **d-g**, UMAPs showing expression of selected marker genes in each cell, including pan-neuronal markers (**d**) and markers for interneurons (**e**), CPN (**f**), and CThPN (**g**). The majority of cells express markers corresponding

to the expected pyramidal neuron subtypes, with only minor contribution of interneurons. **h**, UMAPs showing number of UMIs and number of genes detected in each cell.

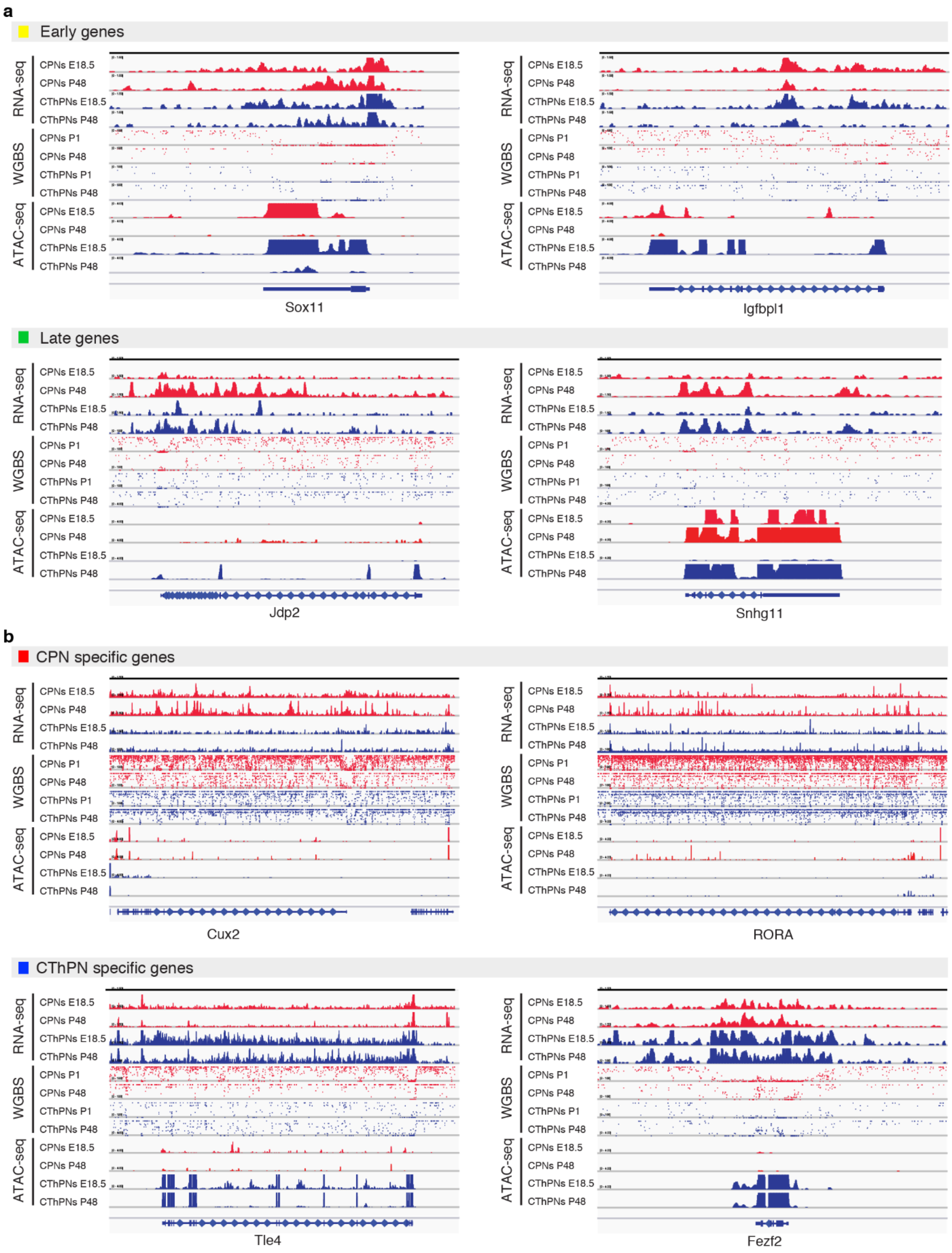

a
Early genes
RNA-seq
WGBS
ATAC-seq
CPNs E18.5
CPNs P48
CThPNs E18.5
CThPNs P48
CPNs P1
CPNs P48
CThPNs P1
CThPNs P48
Sox11
Igfbpl1
Late genes
Jdp2
Snhg11
b
CPN specific genes
Cux2
RORA
CThPN specific genes
Tle4
Fezf2

**Supplementary Fig. 2. Example gene tracks for genes classified into different transcriptional categories.** Examples showing Genome Browser views of RNA sequencing, ATAC-seq, and WGBS tracks, for examples of genes in (**a**) shared-developmental and (**b**) class-specific categories. Red: Cux2 CPNs; Blue: Tle4 CThPNs.

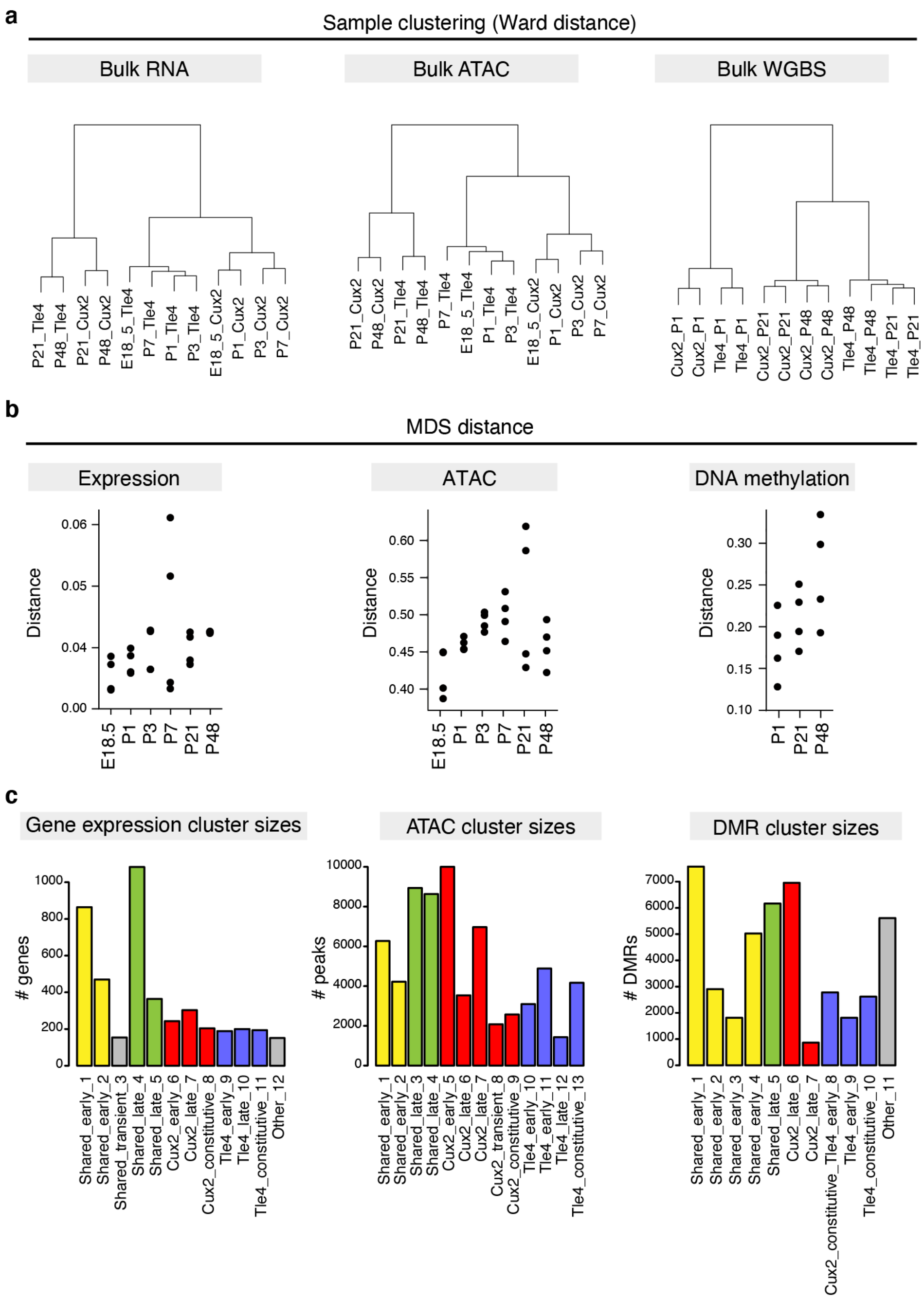


**Supplementary Fig. 3. Characterizing bulk datasets. a**, Dendrograms showing relationships between samples from different ages for each dataset. **b**, Distribution of pairwise sample distances (1-pearson correlation) between replicates at each time point (n = 4 independent biological samples per timepoint), for gene expression, open chromatin and DNAme. **c**, Number of features (genes, ATAC peaks, or DMRs) in each cluster for each dataset.

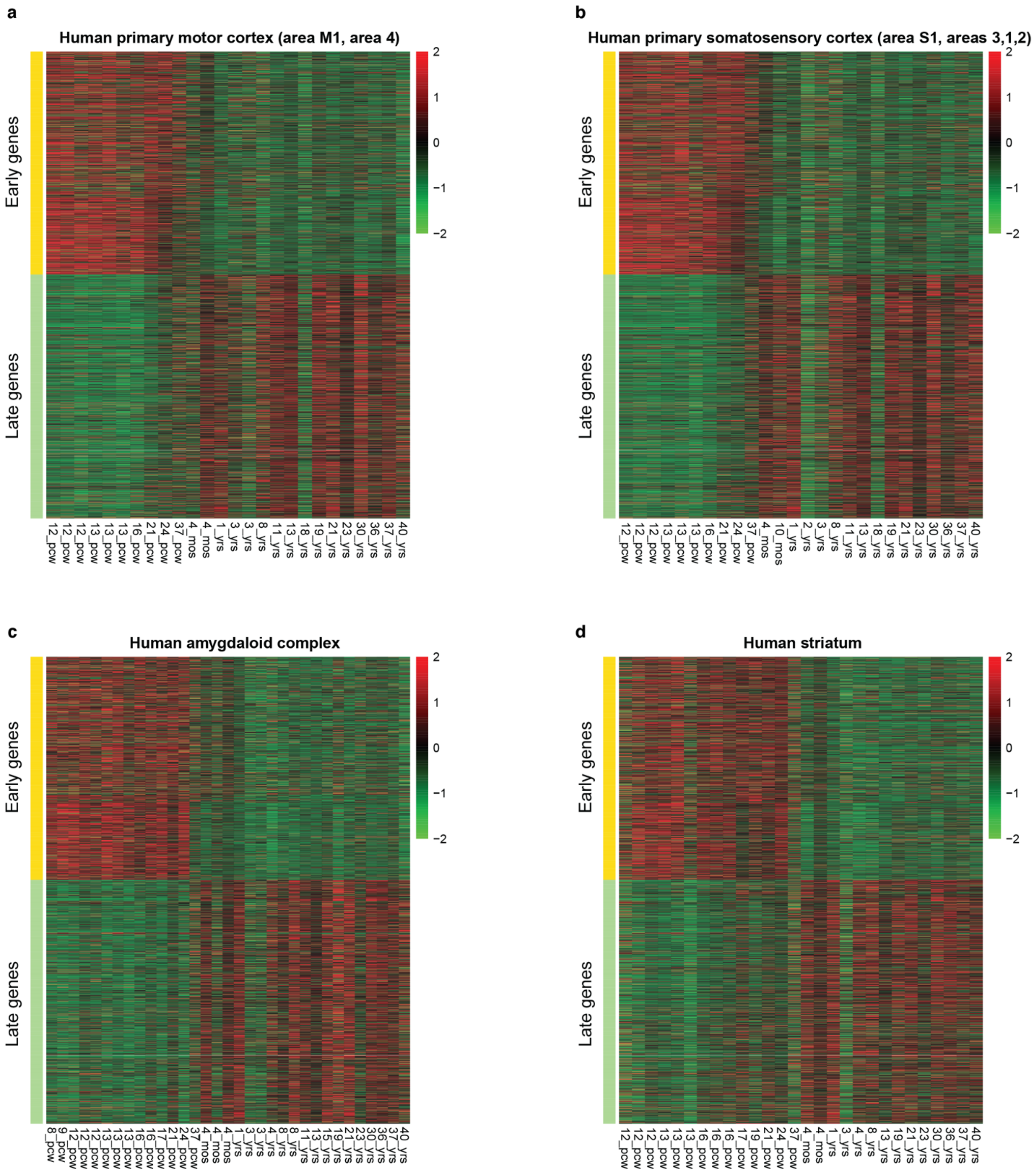


**Supplementary Fig. 4. Validation of temporal patterns of gene expression using external datasets. a-d**, Expression of the human orthologs of the genes in the shared-early and shared-late gene clusters in human developing brain transcriptomic data from BrainSpan. Expression is shown at ages from 8 (amygdaloid complex) or 12 (all others) post-conceptional weeks to 40 years, for four brain regions: primary motor cortex (**a**), primary somatosensory cortex (**b**), amygdaloid

complex (**c**), and striatum (**d**). Color scale: row z-score. Abbreviations: pcw, post-conceptional weeks; mos, months; yrs, years.

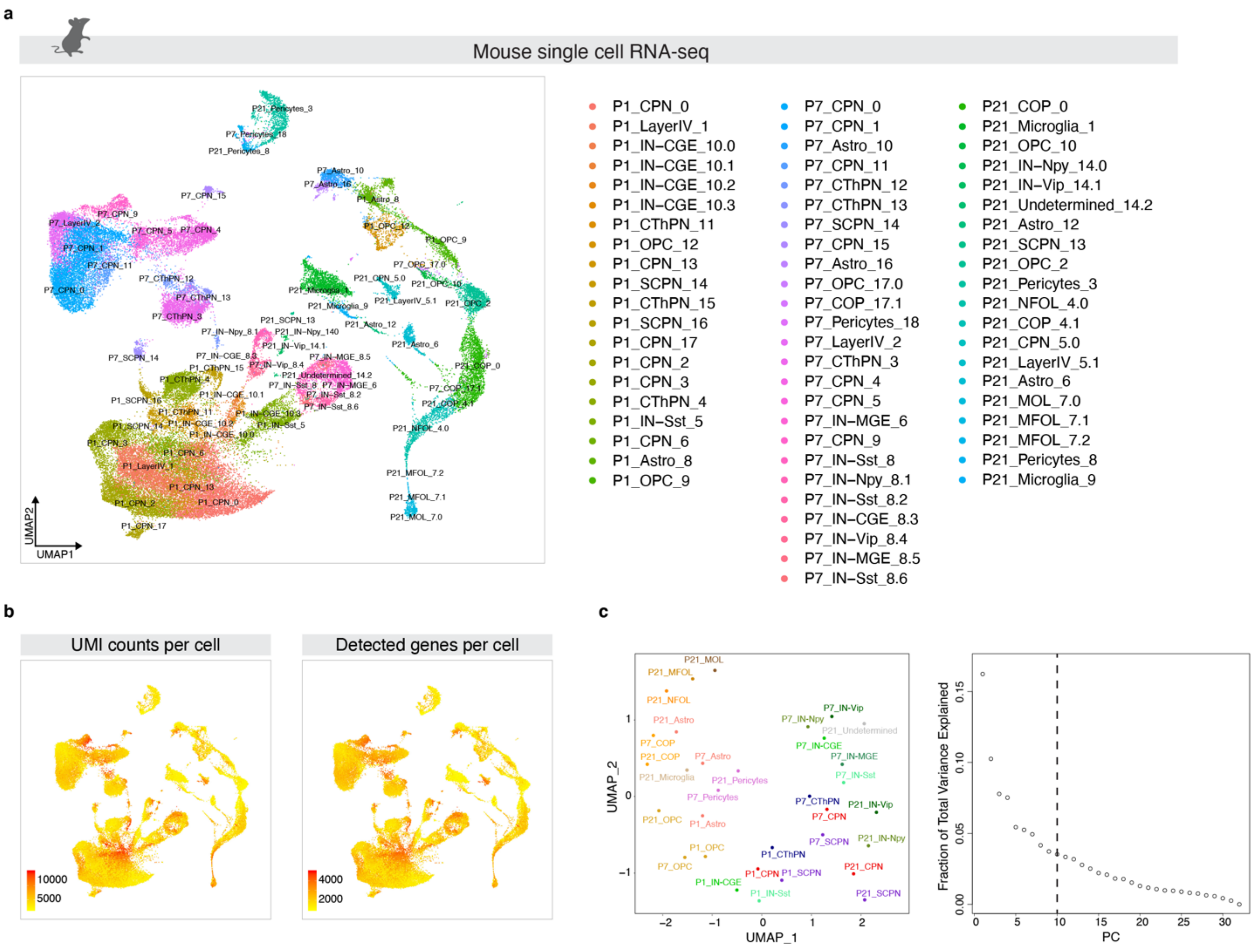


**Supplementary Fig. 5. Characterization of mouse unfractionated single-cell RNA-seq dataset. a**, UMAPs showing each cell cluster, broken down by age. **b**, UMAPs showing number of UMIs and number of genes detected in each cell. **c**, Similarity between cell clusters, as a UMAP representation of the top 10 principal components (PCs) after PCA computed on the average gene expression space.

a

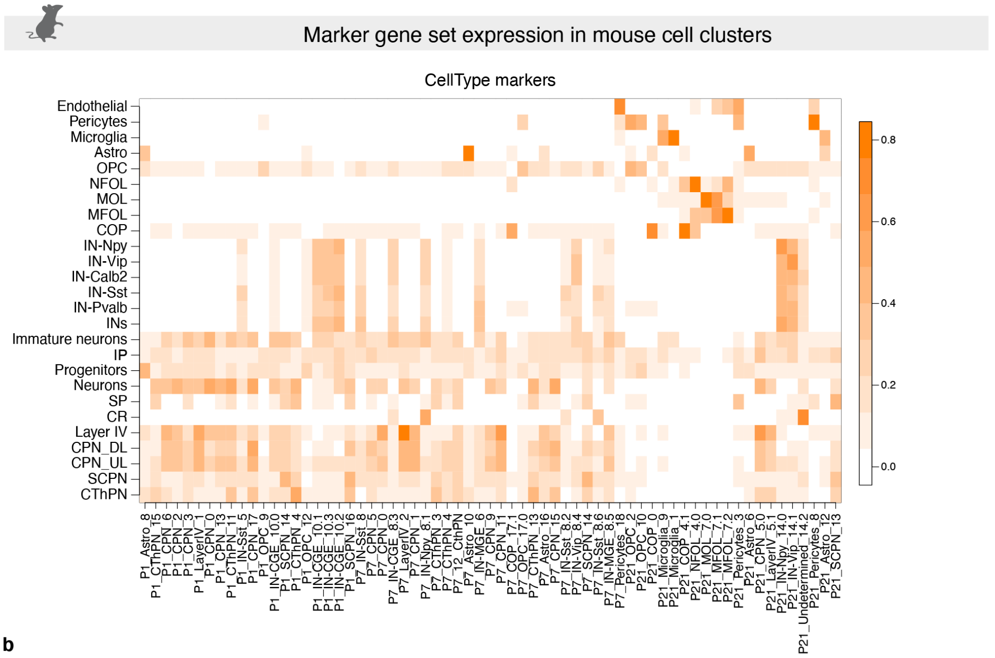
Marker gene set expression in mouse cell clusters
CellType markers
Endothelial
Pericytes
Microglia
Astro
OPC
NFOL
MOL
MFOL
COP
IN-Npy
IN-Vip
IN-Calb2
IN-Sst
IN-Pvalb
INs
Immature neurons
IP
Progenitors
Neurons
SP
CR
Layer IV
CPN_DL
CPN_UL
SCPN
CThPN
0.8
0.6
0.4
0.2
0.0

b

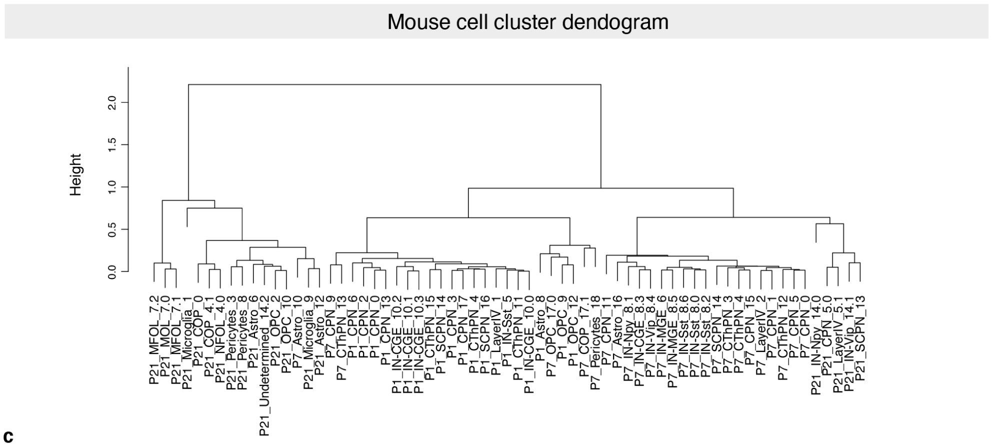
Mouse cell cluster dendogram
Height
0.0
0.5
1.0
1.5
2.0

c

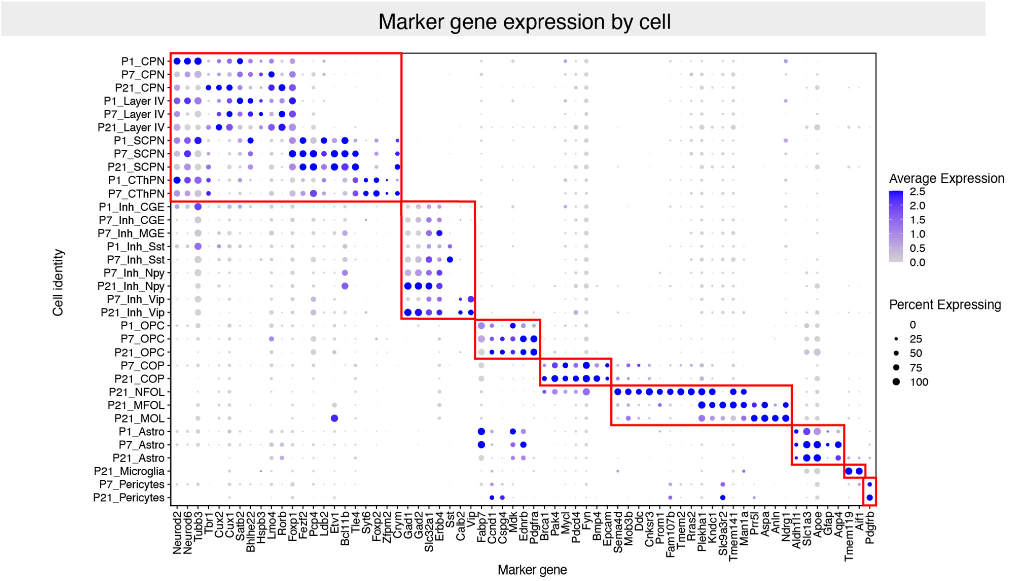
Marker gene expression by cell
Cell identity
Marker gene
Average Expression
2.5
2.0
1.5
1.0
0.5
0.0
Percent Expressing
0
25
50
75
100

**Supplementary Fig. 6. Characterization of mouse unfractionated single-cell RNA-seq dataset. a**, Expression of gene sets representing combined expression of panels of known cell-type marker genes for each cell cluster. **b**, Dendrogram representing similarity between cell clusters. **c**, Average expression and percent of cells expressing for selected marker genes within cells of each assigned cell identity.

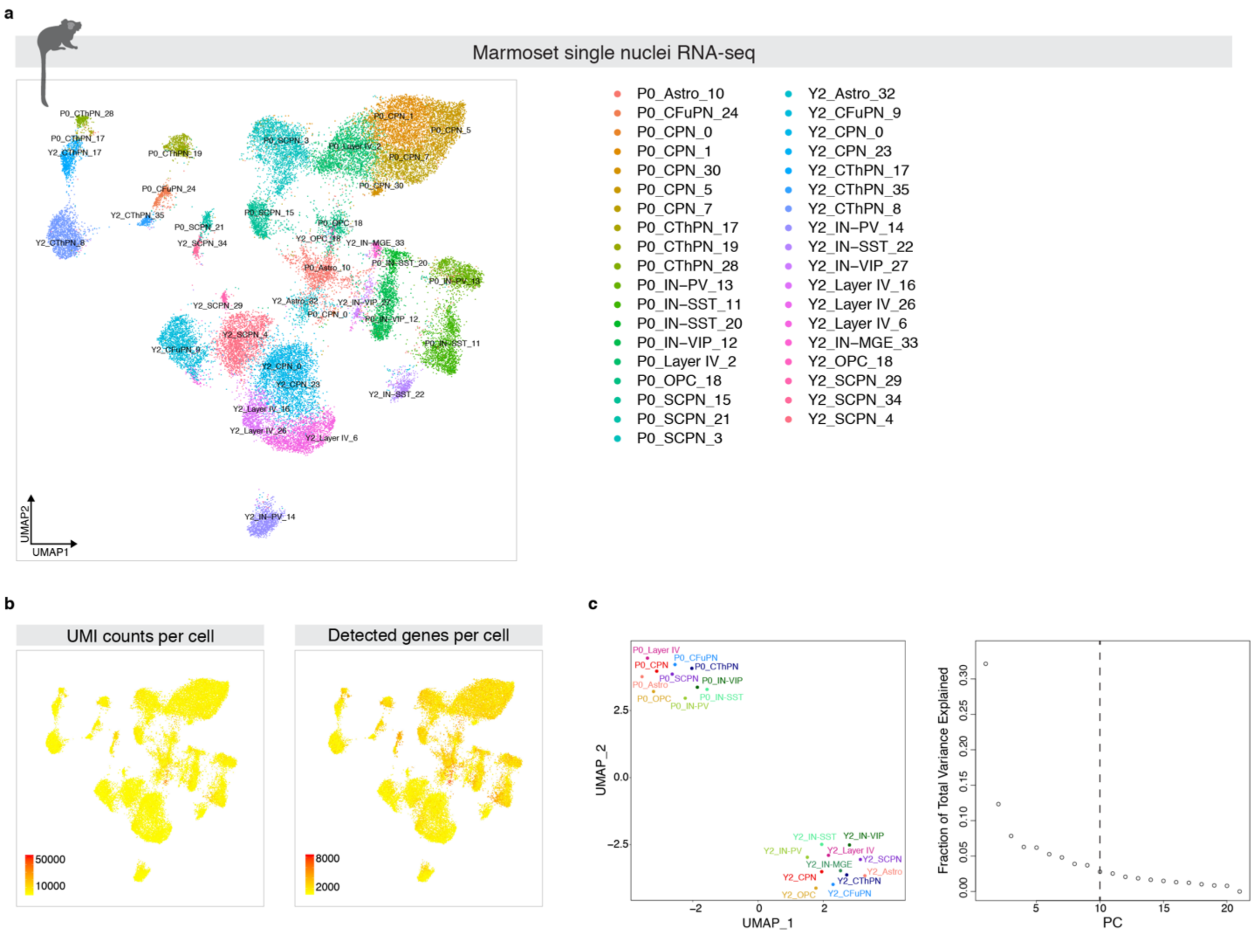


**Supplementary Fig. 7. Characterization of marmoset single-cell RNA-seq dataset. a**, UMAPs showing each cell cluster, broken down by age. **b**, UMAPs showing number of UMIs and number of genes detected in each cell. **c**, Similarity between cell clusters, as a UMAP representation of the top 10 principal components (PCs) after PCA computed on the average gene expression space.

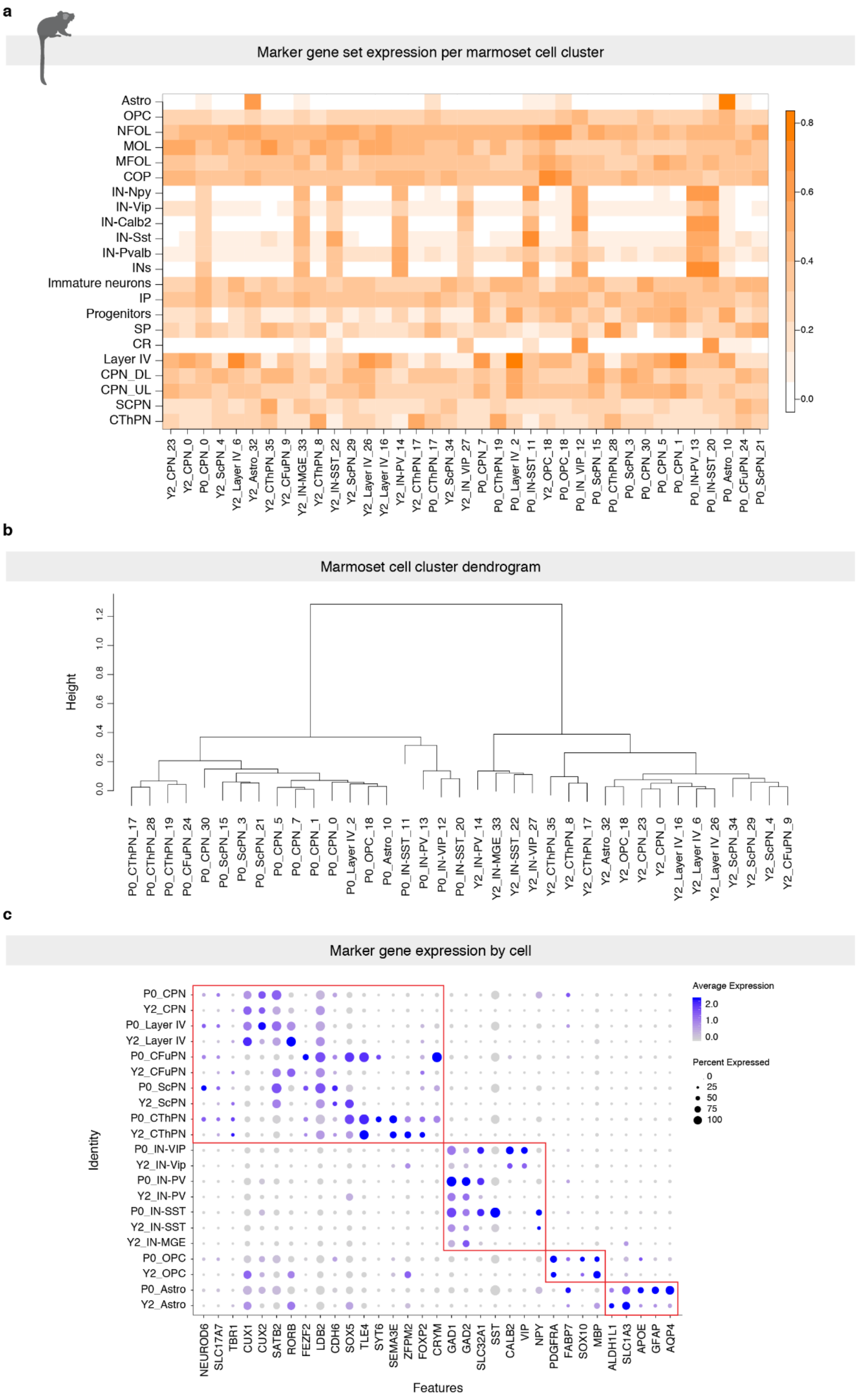

a
Marker gene set expression per marmoset cell cluster
Astro
OPC
NFOL
MOL
MFOL
COP
IN-Npy
IN-Vip
IN-Calb2
IN-Sst
IN-Pvalb
INs
Immature neurons
IP
Progenitors
SP
CR
Layer IV
CPN_DL
CPN_UL
SCPN
CThPN
0.8
0.6
0.4
0.2
0.0
Y2_CPN_23 Y2_CPN_0 P0_CPN_0 Y2_ScPN_4 Y2_Layer IV_6 Y2_Astro_32 Y2_CThPN_35 Y2_CFuPN_9 Y2_IN-MGE_33 Y2_CThPN_8 Y2_IN-SST_22 Y2_ScPN_29 Y2_Layer IV_26 Y2_Layer IV_16 Y2_IN-PV_14 Y2_CThPN_17 P0_CThPN_17 Y2_ScPN_34 Y2_IN_VIP_27 P0_CPN_7 P0_CThPN_19 P0_Layer IV_2 P0_IN-SST_11 Y2_OPC_18 P0_OPC_18 P0_IN_VIP_12 P0_ScPN_15 P0_CThPN_28 P0_ScPN_3 P0_CPN_30 P0_CPN_5 P0_CPN_1 P0_IN-PV_13 P0_IN-SST_20 P0_Astro_10 P0_CFuPN_24 P0_ScPN_21
b
Marmoset cell cluster dendrogram
Height
0.0 0.2 0.4 0.6 0.8 1.0 1.2
P0_CThPN_17 P0_CThPN_28 P0_CThPN_19 P0_CFuPN_24 P0_CPN_30 P0_ScPN_15 P0_ScPN_3 P0_ScPN_21 P0_CPN_5 P0_CPN_7 P0_CPN_1 P0_CPN_0 P0_Layer IV_2 P0_OPC_18 P0_Astro_10 P0_IN-SST_11 P0_IN-PV_13 P0_IN-VIP_12 P0_IN-SST_20 Y2_IN-PV_14 Y2_IN-MGE_33 Y2_IN-SST_22 Y2_IN-VIP_27 Y2_CThPN_35 Y2_CThPN_8 Y2_CThPN_17 Y2_Astro_32 Y2_OPC_18 Y2_CPN_23 Y2_CPN_0 Y2_Layer IV_16 Y2_Layer IV_6 Y2_Layer IV_26 Y2_ScPN_34 Y2_ScPN_29 Y2_ScPN_4 Y2_CFuPN_9
c
Marker gene expression by cell
Identity
P0_CPN
Y2_CPN
P0_Layer IV
Y2_Layer IV
P0_CFuPN
Y2_CFuPN
P0_ScPN
Y2_ScPN
P0_CThPN
Y2_CThPN
P0_IN-VIP
Y2_IN-Vip
P0_IN-PV
Y2_IN-PV
P0_IN-SST
Y2_IN-SST
Y2_IN-MGE
P0_OPC
Y2_OPC
P0_Astro
Y2_Astro
NEUROD6 SLC17A7 TBR1 CUX1 CUX2 SATB2 RORB FEZF2 LDB2 CDH6 SOX5 TLE4 SYT6 SEMA3E ZFPM2 FOXP2 CRYM GAD1 GAD2 SLC32A1 SST CALB2 VIP NPY PDGFRA FABP7 SOX10 MBP ALDH1L1 SLC1A3 APOE GFAP AQP4
Features
Average Expression
2.0
1.0
0.0
Percent Expressed
0
25
50
75
100

**Supplementary Fig. 8. Characterization of marmoset single-cell RNA-seq dataset. a**, Expression of gene sets representing combined expression of panels of known cell-type marker genes for each cell cluster. **b**, Dendrogram representing similarity between cell clusters. **c**, Average expression and percent of cells expressing for selected marker genes within cells of each assigned cell identity.

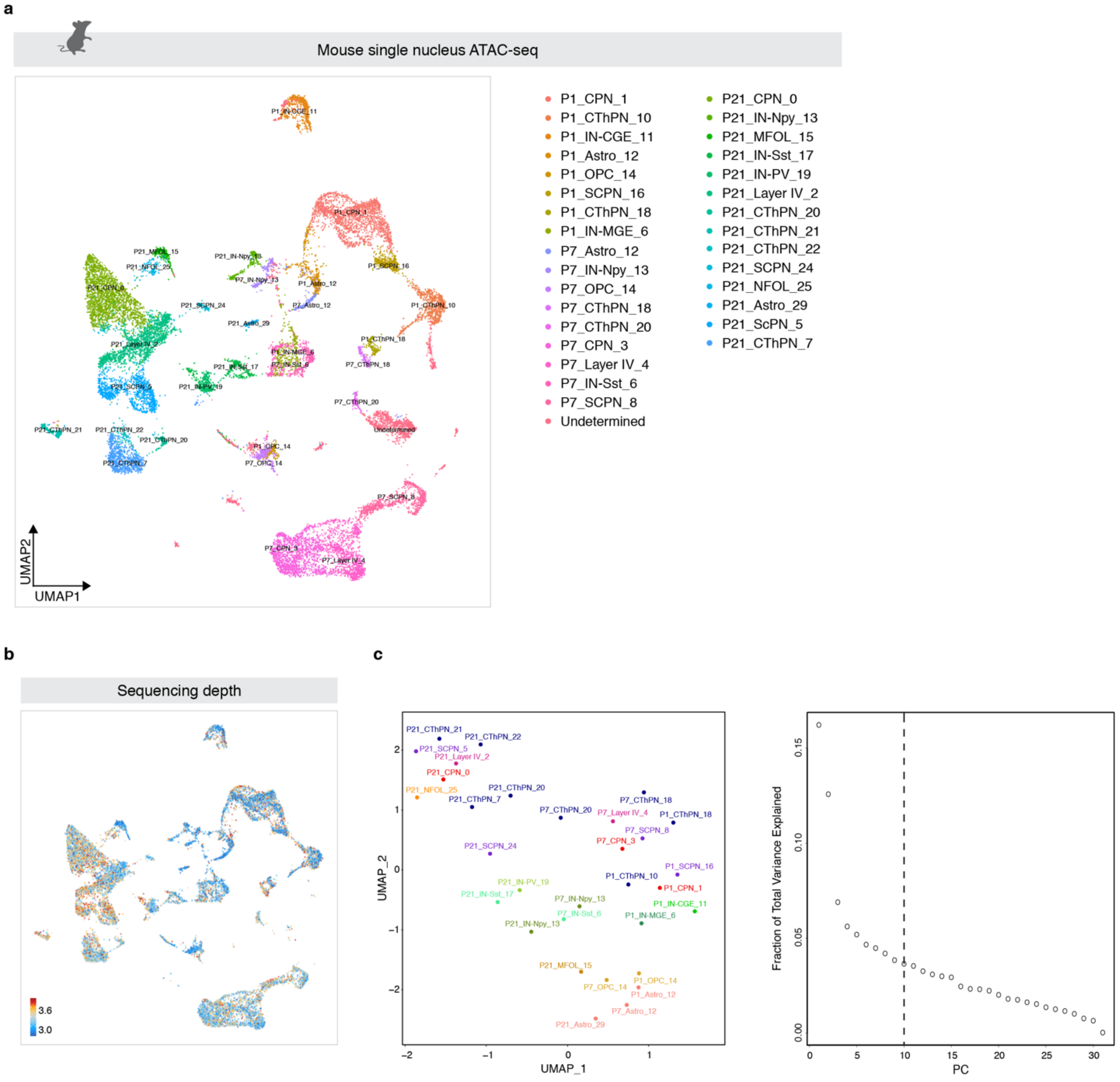


**Supplementary Fig. 9. Characterization of mouse single-cell ATAC-seq dataset. a**, UMAPs showing each cell cluster, broken down by age. **b**, UMAP showing depth of sequencing for each cell. **c**, Similarity between cell clusters, as a UMAP representation of the top 10 principal components (PCs) after PCA computed on the average gene expression space.

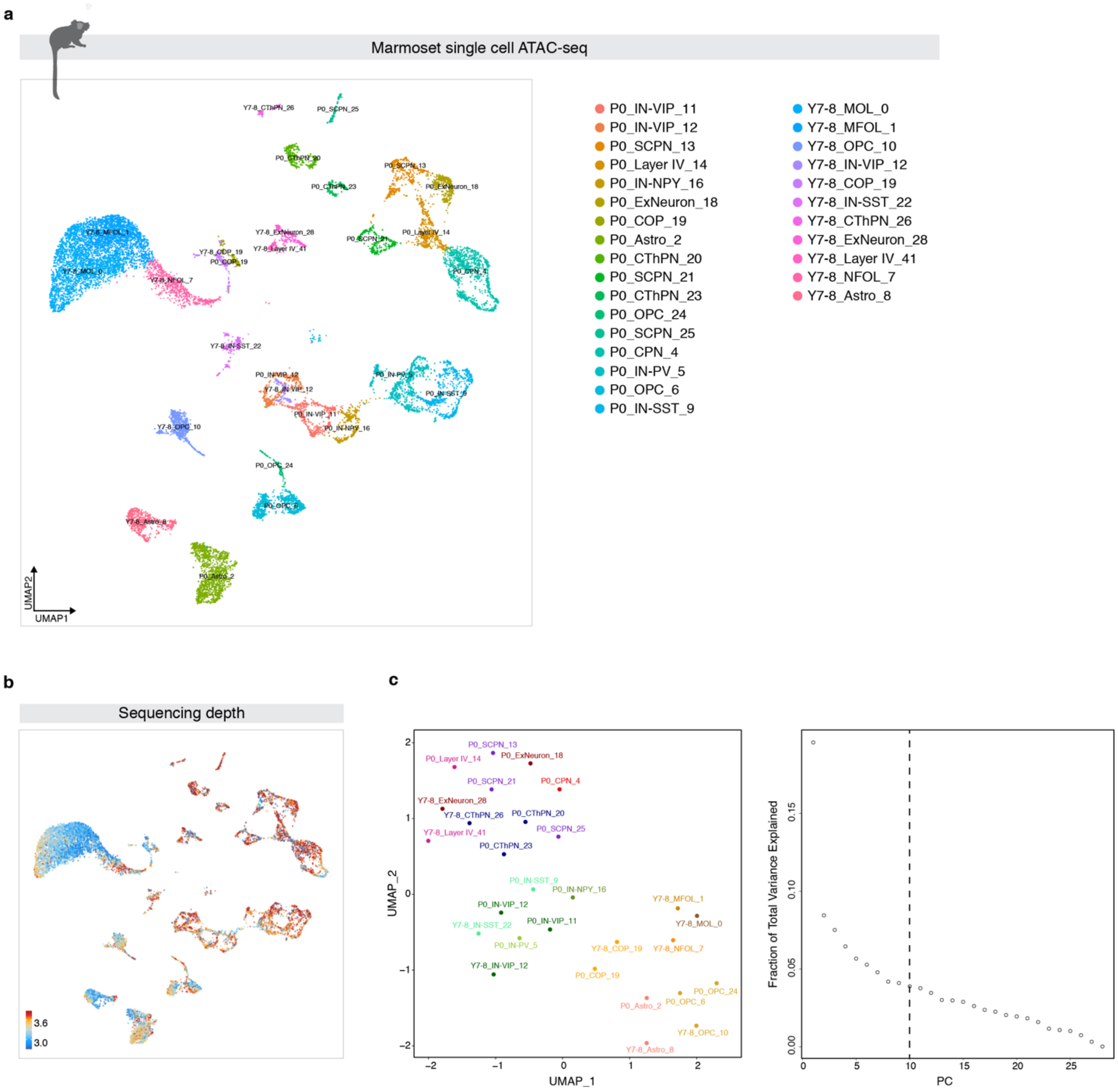


**Supplementary Fig. 10. Characterization of marmoset single-cell ATAC-seq dataset. a**, UMAPs showing each cell cluster, broken down by age. **b**, UMAP showing depth of sequencing for each cell. **c**, Similarity between cell clusters, as a UMAP representation of the top 10 principal components (PCs) after PCA computed on the average gene expression space.

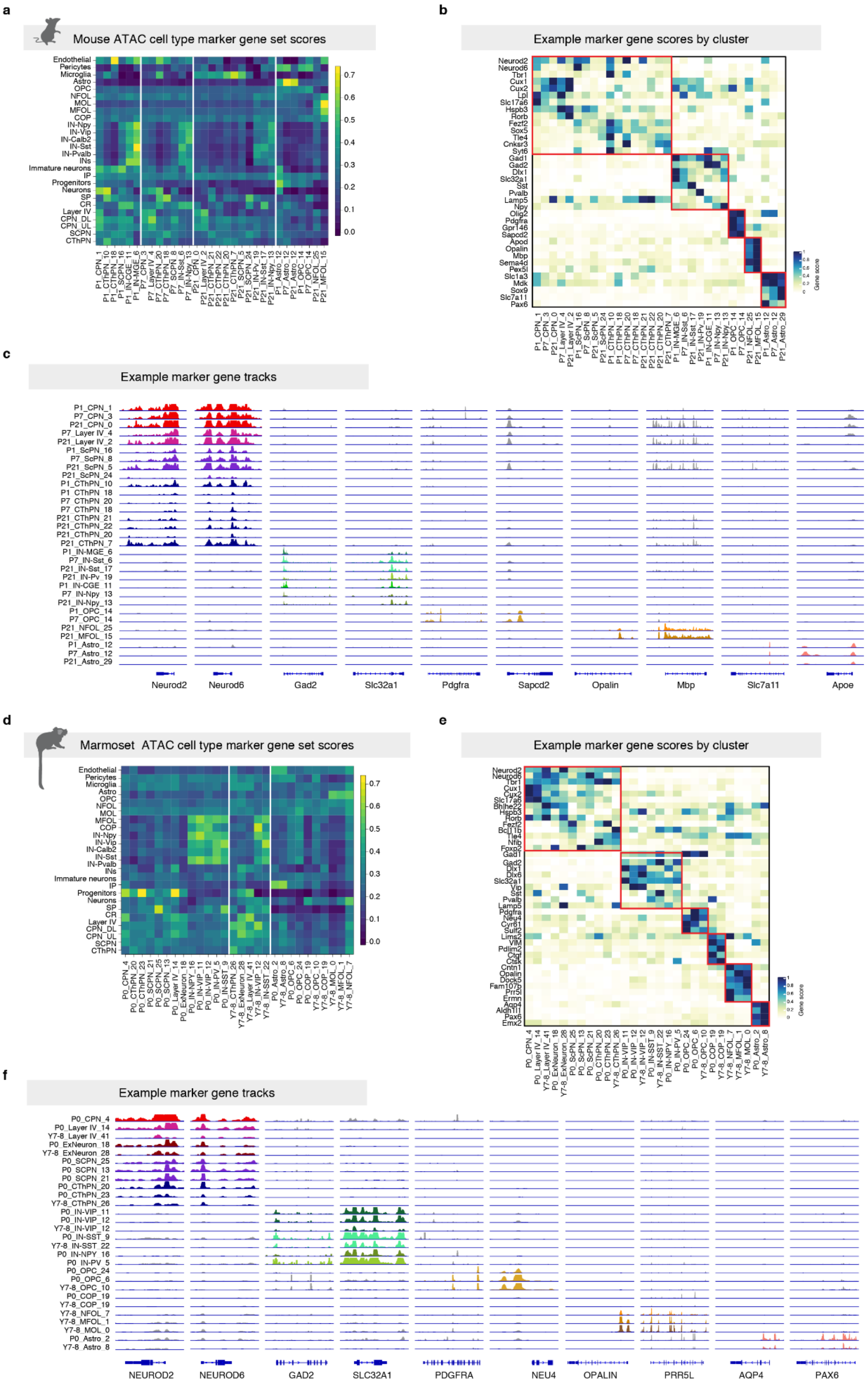
a
Mouse ATAC cell type marker gene set scores
b
Example marker gene scores by cluster
c
Example marker gene tracks
Neurod2
Neurod6
Gad2
Slc32a1
Pdgfra
Sapcd2
Opalin
Mbp
Slc7a11
Apoe
d
Marmoset ATAC cell type marker gene set scores
e
Example marker gene scores by cluster
f
Example marker gene tracks
NEUROD2
NEUROD6
GAD2
SLC32A1
PDGFRA
NEU4
OPALIN
PRR5L
AQP4
PAX6

**Supplementary Fig. 11. Cell-type assignments for scATAC datasets. a**, Average gene score for mouse assigned cell identities using the same panel of cell-type marker gene sets as in Supplementary Fig. 5. **b**, Gene score for example individual marker genes. **c**, Mouse gene tracks for example marker genes across each assigned cell identity. **d**, Average gene score for marmoset assigned cell identities using the same panels of cell-type marker gene sets as in Supplementary Fig. 7. **e**, Gene score for example individual marker genes. **f**, Marmoset gene tracks for example marker genes across each assigned cell identity.

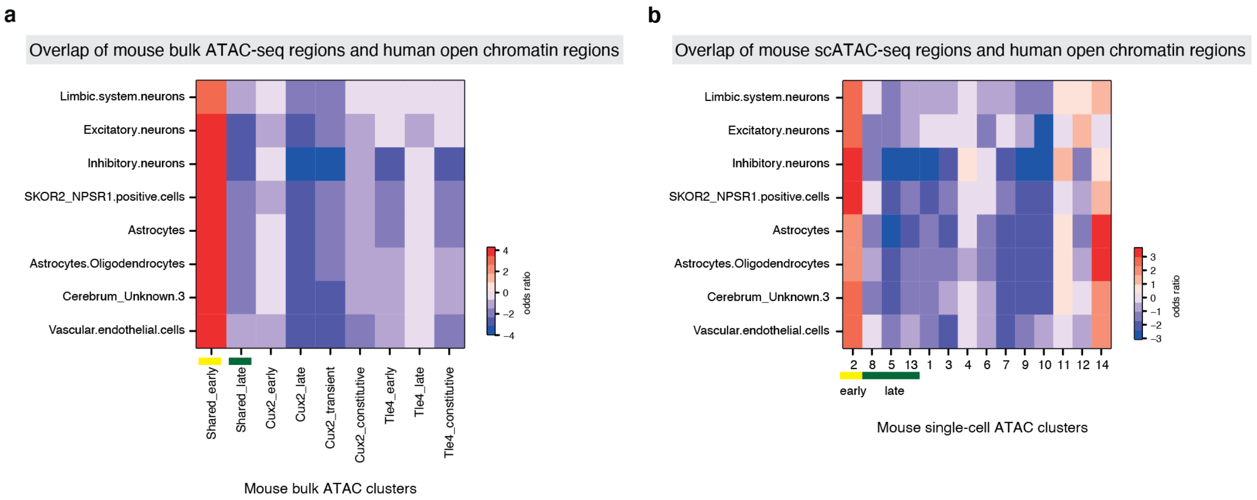


**Supplementary Fig. 12. Correlation of mouse versus human chromatin open regions. a**, Odds ratio for significant (BH corrected p-value <=0.05) overlaps of mouse bulk ATAC-seq clusters for PN (x-axis) and open chromatin regions in human fetal cerebrum. **b**, Odds ratio for significant (BH corrected p-value <=0.05) overlaps of mouse neuronal scATAC-seq-based open chromatin clusters (shown in e) (x-axis) and open chromatin regions in human fetal cerebrum.
