# Supplementary material for: Temporally divergent regulatory mechanisms govern neuronal diversification and maturation in the mouse and marmoset neocortex
Source: Nat Neurosci. 2022 Aug 1;25(8):1049–58. doi: 10.1038/s41593-022-01123-4 (PMC9343253; doi:10.1038/s41593-022-01123-4)
Supplement: Supplementary file 2 — Reporting Summary [file 41593_2022_1123_MOESM2_ESM.pdf]

nature research

Corresponding author(s): Paola Arlotta, Michael Ziller

Last updated by author(s): Jun 7, 2022

Reporting Summary

Nature Research wishes to improve the reproducibility of the work that we publish. This form provides structure for consistency and transparency in reporting. For further information on Nature Research policies, see our Editorial Policies and the Editorial Policy Checklist.

Statistics

For all statistical analyses, confirm that the following items are present in the figure legend, table legend, main text, or Methods section.

n/a	Confirmed	
☐	☒	The exact sample size (*n*) for each experimental group/condition, given as a discrete number and unit of measurement
☒	☐	A statement on whether measurements were taken from distinct samples or whether the same sample was measured repeatedly
☐	☒	The statistical test(s) used AND whether they are one- or two-sided<br>*Only common tests should be described solely by name; describe more complex techniques in the Methods section.*
☒	☐	A description of all covariates tested
☐	☒	A description of any assumptions or corrections, such as tests of normality and adjustment for multiple comparisons
☒	☐	A full description of the statistical parameters including central tendency (e.g. means) or other basic estimates (e.g. regression coefficient) AND variation (e.g. standard deviation) or associated estimates of uncertainty (e.g. confidence intervals)
☐	☒	For null hypothesis testing, the test statistic (e.g. *F*, *t*, *r*) with confidence intervals, effect sizes, degrees of freedom and *P* value noted<br>*Give P values as exact values whenever suitable.*
☒	☐	For Bayesian analysis, information on the choice of priors and Markov chain Monte Carlo settings
☒	☐	For hierarchical and complex designs, identification of the appropriate level for tests and full reporting of outcomes
☒	☐	Estimates of effect sizes (e.g. Cohen's *d*, Pearson's *r*), indicating how they were calculated

Our web collection on statistics for biologists contains articles on many of the points above.

Software and code

Policy information about availability of computer code

Data collection	Volocity image analysis software v4.0.1
Data analysis	Custom scripts used in this study are available from the corresponding authors on request.<br><br>The following programs were used:<br><br>Cell Ranger version 3.0.1<br>Trimmomatic version 0.33<br>TopHat2 version 2.0.13<br>Cuffdiff version 2.2.1<br>Bowtie2 version 2.3.2<br>Picard toolkit version 2.7.1<br>MACS2 version 2.1.1<br>MOABS version 1.3.2<br>SAMtools version 1.5<br>sequtils version 1.74<br>bcl2fastq<br>R package DSS version 2.14<br>methylKit version 0.9.5<br>GREAT version 3.0.0<br>R version 3.5.1<br>R package Seurat version 2.0.0 and 3.1.5<br>R package cluster 2.0.7

R package ggplot2 version 2.3.3
R package cicero version 1.0.15
R package IRanges
liftOver (UCSC command-line liftover tool)
MEME suite version 4.11.2
Scasat
bx-python version 0.8.0

For manuscripts utilizing custom algorithms or software that are central to the research but not yet described in published literature, software must be made available to editors and reviewers. We strongly encourage code deposition in a community repository (e.g. GitHub). See the Nature Research guidelines for submitting code & software for further information.

Data

Policy information about availability of data

All manuscripts must include a data availability statement. This statement should provide the following information, where applicable:

- Accession codes, unique identifiers, or web links for publicly available datasets
- A list of figures that have associated raw data
- A description of any restrictions on data availability

The datasets generated in the current study are available through the Gene Expression Omnibus (GEO SuperSeries GSE204851).

The following publicly-available datasets were used:
Mus musculus ENSEMBL genome build NCBIM37 (mm9); Callithrix jacchus (common marmoset) genome assembly version ASM275486v1.93; Human developing brain transcriptomic data from BrainSpan (brainspan.org/rnaseq/search/index.html); mouse enhancer data from the VISTA enhancer database (enhancer.lbl.gov); mouse CAGE-tag data from the FANTOM5 database (fantom.gsc.riken.jp/5/); mouse ISH expression data from the Allen Brain Atlas Developing Mouse Brain database (developingmouse.brain-map.org); mouse single-cell ATAC-seq data from Cusanovich et al. 2018 (GEO: GSE111586); DNAse HS I peak tracks from the mouse ENCODE consortium (www.encodeproject.org); human fetal cerebrum single-cell ATAC-seq data from Domcke et al. 2020 (descartes.brotmanbaty.org).

Field-specific reporting

Please select the one below that is the best fit for your research. If you are not sure, read the appropriate sections before making your selection.

☒ Life sciences ☐ Behavioural & social sciences ☐ Ecological, evolutionary & environmental sciences

For a reference copy of the document with all sections, see nature.com/documents/nr-reporting-summary-flat.pdf

Life sciences study design

All studies must disclose on these points even when the disclosure is negative.

Sample size	No statistical methods were used to pre-determine sample sizes, but our sample sizes are similar to those reported in previous publications (Ding, J. et al. Systematic comparison of single-cell and single-nucleus RNA-sequencing methods, Nat. Biotechnol. 2020).
Data exclusions	Quality-control filtering for sequencing data was performed using standard metrics as described in the Methods.
Replication	All experiments included at least two biological replicates, and differences between replicates were evaluated. All attempts at replication were succesful.
Randomization	For mouse experiments, animals were randomized during sample collection where possible. For marmoset experiments, sample selection was dictated by tissue availability; all tissue was collected from healthy, un-manipulated individuals.
Blinding	No blinding was performed as the identity of the samples was central to the analysis.

Reporting for specific materials, systems and methods

We require information from authors about some types of materials, experimental systems and methods used in many studies. Here, indicate whether each material, system or method listed is relevant to your study. If you are not sure if a list item applies to your research, read the appropriate section before selecting a response.

Materials & experimental systems

n/a	Involved in the study
☐	☒ Antibodies
☒	☐ Eukaryotic cell lines
☒	☐ Palaeontology and archaeology
☐	☒ Animals and other organisms
☒	☐ Human research participants
☒	☐ Clinical data
☒	☐ Dual use research of concern

Methods

n/a	Involved in the study
☒	☐ ChIP-seq
☐	☒ Flow cytometry
☒	☐ MRI-based neuroimaging

Antibodies

Antibodies used

rat anti-CTIP2 antibody, 1:1,000 (Abcam ab18465)
rabbit anti-CUX1 (CDP M-222), 1:100 (Santa Cruz sc-13024)
mouse anti-SATB2, 1:50 (Abcam ab51502)
Goat anti-rat Alexa 488, 1:1000 (ThermoFisher A48262)
Donkey anti-mouse Alexa 647, 1:1000 (ThermoFisher A-31571)
Donkey anti-rabbit Alexa 647, 1:1000 (Thermo-Fisher A-31573)

Validation

Validation data for Abcam ab51502 is available on the manufacturer's website (https://www.abcam.com/satb2-antibody-satba4b10-c-terminal-ab51502.html), for immunocytochemistry (HT10180 cells), Western blot (NIH/3T3 and HT1080 whole cell lysates), and immunoprecipitation (HeLa cell lysate).

Validation data for Abcam ab18465 is available on the manufacturer's website (https://www.abcam.com/ctip2-antibody-25b6-ab18465.html), for flow cytometry (Jurkat cells), immunocytochemistry (neonatal mouse hippocampal cultured neurons), and Western blot (Jurkat cell nuclear extract; mouse brain tissue lysate).

Validation data for Santa Cruz sc-13024 is available in the manufacturer's product datasheet (https://datasheets.scbt.com/sc-13024.pdf), for Western blot (K-562 and BJAB nuclear extracts) and immunocytochemistry (HeLa cells and human urinary bladder tissue).

Animals and other organisms

Policy information about studies involving animals; ARRIVE guidelines recommended for reporting animal research

Laboratory animals

Mouse, male and female, E18.5 to P48; C57BL/6J (JAX 000664) WT, tamoxifen-inducible tdTomato reporter Ai14 (JAX 007914) heterozygous, Cux2-CreERT2 (MMRRC 032779-MU) heterozygous, and Tle4-2A-CreERT2 (JAX 036298) heterozygous.

Wild animals

No wild animals were used in the study.

Field-collected samples

No field-collected samples were used in the study.

Ethics oversight

Experiments using mice were conducted under protocols approved by the Harvard University Institutional Animal Care and Use Committee and followed the guidelines set forth in the National Institute of Health Guide for the Care and Use of Laboratory Animals. All marmoset experiments were approved by the Institutional Animal Care and Use Committee of Massachusetts Institute of Technology and followed the guidelines from the National Institute of Health's Guide for the Care and Use of Laboratory Animals.

Note that full information on the approval of the study protocol must also be provided in the manuscript.

Flow Cytometry

Plots

Confirm that:

☒ The axis labels state the marker and fluorochrome used (e.g. CD4-FITC).

☒ The axis scales are clearly visible. Include numbers along axes only for bottom left plot of group (a 'group' is an analysis of identical markers).

☒ All plots are contour plots with outliers or pseudocolor plots.

☒ A numerical value for number of cells or percentage (with statistics) is provided.

Methodology

Sample preparation

Fresh mouse somatosensory and motor cortex was dissociated by papain digestion at 37°C in medium containing 0.8 mM kynurenic acid and 0.05 mM APV. Digestion was stopped with room-temperature medium containing 10 mg/ml each of ovomucoid protease inhibitor and BSA, and tissue was mechanically dissociated by gentle trituration in ice-cold medium with

0.4 mM kynurenic acid and 0.025 mM APV. For full details, see Methods.

Instrument	BD FACSAria II+
Software	BD FACSDiva, FloJo
Cell population abundance	Abundance was between 0.5% and 8.5%, depending on sample. Purity was evaluated by fluorescence imaging of sorted cells (Extended Data Fig 1), and by single-cell sequencing of sorted populations (Supplementary Fig 1).
Gating strategy	DAPI-negative, Vybrant DyeCycle Ruby-positive events were gated on tdTomato fluorescence vs GFP-channel autofluorescence as shown in Extended Data Fig 1b.

☒ Tick this box to confirm that a figure exemplifying the gating strategy is provided in the Supplementary Information.
